# Glutamatergic signaling and low prodynorphin expression are associated with intact memory and reduced anxiety in rat models of healthy aging

**DOI:** 10.3389/fnagi.2014.00081

**Published:** 2014-05-07

**Authors:** Caroline Ménard, Rémi Quirion, Sylvain Bouchard, Guylaine Ferland, Pierrette Gaudreau

**Affiliations:** ^1^Neuroscience Division, Douglas Mental Health University Institute Research CenterMontreal, QC, Canada; ^2^Department of Psychiatry, McGill UniversityMontreal, QC, Canada; ^3^Laboratory of Neuroendocrinology of Aging, Centre Hospitalier de l'Université de Montréal Research CenterMontreal, QC, Canada; ^4^Department of Medicine, University of MontrealMontreal, QC, Canada; ^5^Faculty of Medicine, University of MontrealMontreal, QC, Canada; ^6^Hôpital du Sacré-Coeur de Montréal Research CenterMontreal, QC, Canada; ^7^Department of Nutrition, University of MontrealMontreal, QC, Canada

**Keywords:** aging, cognition, anxiety, caloric restriction, obesity, glutamate receptors

## Abstract

The LOU/C/Jall (LOU) rat strain is considered a model of healthy aging due to its increased longevity, maintenance of stable body weight (BW) throughout life and low incidence of age-related diseases. However, aging LOU rat cognitive and anxiety status has yet to be investigated. In the present study, male and female LOU rat cognitive performances (6–42 months) were assessed using novel object recognition and Morris Water Maze tasks. Recognition memory remained intact in all LOU rats up to 42 months of age. As for spatial memory, old LOU rat performed similarly as young animals for learning acquisition, reversal learning, and retention. While LOU rat BW remained stable despite aging, 20-month-old *ad-libitum*-fed (OAL) male Sprague Dawley rats become obese. We determined if long-term caloric restriction (LTCR) prevents age-related BW increase and cognitive deficits in this rat strain, as observed in the obesity-resistant LOU rats. Compared to young animals, recognition memory was impaired in OAL but intact in 20-month-old calorie-restricted (OCR) rats. Similarly, OAL spatial learning acquisition was impaired but LTCR prevented the deficits. Exacerbated stress responses may favor age-related cognitive decline. In the elevated plus maze and open field tasks, LOU and OCR rats exhibited high levels of exploratory activity whereas OAL rats displayed anxious behaviors. Expression of prodynorphin (Pdyn), an endogenous peptide involved in stress-related memory impairments, was increased in the hippocampus of OAL rats. Group 1 metabotropic glutamate receptor 5 and immediate early genes *Homer 1a* and *Arc* expression, both associated with successful cognitive aging, were unaltered in aging LOU rats but lower in OAL than OCR rats. Altogether, our results, supported by principal component analysis and correlation matrix, suggest that intact memory and low anxiety are associated with glutamatergic signaling and low Pdyn expression in the hippocampus of non-obese aging rats.

## Introduction

Chronic conditions such as diabetes and obesity have become a growing concern in public health around the world and its prevalence is rising in different age groups, including older adults (Mathus-Vliegen, 2012). High fat and calorie intakes not only lead to metabolic disorders but could also accelerate age-related cognitive decline (Williamson et al., 2012; Fadel et al., 2013). Epidemiological studies suggest that type 2 diabetes and obesity increase the risk of dementias (Williamson et al., 2012). Stress exposure over the lifespan may also accelerate cellular aging and promote cognitive dysfunction (Lupien et al., 2009; O'Donovan et al., 2013).

Animal models of healthy aging could help increase our understanding of the impact of obesity on cellular and molecular processes underlying cognitive dysfunctions and anxious status. The LOU/C/Jall (LOU) rat strain is characterized by increased longevity (median lifespan of 29 and 34 months for males and females, respectively), a low and stable adipose tissue mass and a reduced incidence of common age-related diseases (Alliot et al., 2002). To date, no study has examined the changes in recognition and spatial memory and anxiety behavioral components in young adult (6 months) to very old (38–42 months) male and female LOU rats. The single neurobehavioral study performed to date on LOU rats, investigating exclusively recognition memory in young and 24-month-old male LOU rat, showed no change with age (Kollen et al., 2010).

Hippocampus-dependent memory involves glutamatergic transmission (Baudry et al., 2011; Menard and Quirion, 2012a). Notably, cognitive decline is characterized by a decrease in glutamate receptor levels altering synaptic plasticity in the aged mouse brain (Zhao et al., 2009). Spatial memory can be rescued when the expression of N-methyl-D-aspartate (NMDA) receptor NR2B subunit is enhanced (Brim et al., 2012). Group 1 metabotropic glutamate receptors (mGluR) were recently correlated with successful cognitive aging in Long–Evans male rats (Menard and Quirion, 2012b; Yang et al., 2013). Their activation plays crucial roles in the formation of both spatial (Lu et al., 1997; Balschun et al., 1999; Menard and Quirion, 2012b) and recognition memory (Barker et al., 2006; Christoffersen et al., 2008) in rodents.

In the present study, the effects of aging and body mass on recognition and spatial memory, anxious behaviors and related synaptic plasticity was investigated using two models of successful aging: the obesity-resistant LOU rat (Alliot et al., 2002) and the Sprague Dawley (SD) rat subjected to long-term caloric restriction (LTCR) (Bedard et al., 2010, 2013; Moyse et al., 2012). LTCR, the most powerful nutritional intervention known to increase longevity in good health, has been associated with enhanced hippocampal plasticity in rodents (Stranahan and Mattson, 2011). Altogether our results indicate that intact memory, glutamate receptor levels, and immediate early gene (IEG) expression were observed in LOU rats up to 42 month-old while age-related memory deficits were partially prevented by LTCR. Synaptic plasticity was altered and anxious behaviors significantly increased in obese old *ad-libitum*-fed (OAL) rats compared to the other groups. It might be linked to higher prodynorphin (Pdyn) expression, a neuropeptide associated with age-related memory impairment and stress-induced disorders (Menard et al., 2013).

## Materials and methods

### Animals

Young adult (Y: 6 months, *n* = 12: 6 males, 6 females), mature (M: 12 months, *n* = 15: 7 males, 8 females), old (O: 24 months, *n* = 12: 7 males, 5 females) and old-old (O/O: 38–42-months, *n* = 13: 3 males, 10 females) LOU rats, and young adult *ad-libitum*-fed (AL) (YAL: 3 months, *n* = 8), old AL (OAL: 20 months, *n* = 5), and old calorie-restricted (CR) (OCR: 20 months, *n* = 11) male SD rats were obtained from the Quebec Network for Research on Aging rat colonies. Sampling size was determined according to a published study comparing novel object recognition performances in 24-month-old LOU and SD rats (Kollen et al., 2010). Rats had free access to chow, except for CR rats, and water. At 7.5 months, SD rats were subjected or not to 20% caloric restriction for 2 weeks and 40% thereafter until death (Bedard et al., 2010, 2013; Moyse et al., 2012). AL and CR SD rats were fed chow Teklad control diet TD04088 and fortified TD 04089, respectively, to ensure that the two groups receive equivalent amounts of minerals and vitamins (Harlan Teklad, Madison, WI), as previously described (Moyse et al., 2012). LOU rats were fed chow A03 SAFE growing diet for 3 weeks after weaning and the maintenance A04 SAFE diet thereafter (Perotech, Toronto, CA) (Veyrat-Durebex et al., 2005). All rats were fed between 07:00 and 08:00 h. SD (1/cage) and LOU (2–3/cage) rats were housed in plastic cages in temperature-, humidity-, and lighting-controlled rooms (12:12-h light-dark cycles; lights on at 07:00 h). Food intake and body weight (BW) were recorded regularly. BW and serum corticosterone (CORT) levels of each group are shown in Supplemental Table 1. To control for possible effects of circadian rhythms, all trials were performed during an established time frame (10:00–15:00). For behavioral experiments, animals were trained in two successive groups. The first included all SD rats and very old male and female LOU rats whereas the second one included young, mature and old male and female LOU rats. Upon completion of behavioral testing, non-fasted unaesthetized rats were quickly sacrificed by rapid decapitation for *ex vivo* analyses (Whittington et al., 2013). Animal care, surgery and handling procedures were approved by the CHUM Research Center Animal Care Committees in compliance with the Canadian Council for Animal Care.

### Novel object recognition (NOR)

This task was used to evaluate episodic and reference memory. The rats were first exposed to the empty arena (41 × 41 × 21 cm DietScan clear plexiglass cage; AccuScan Instruments Inc., Columbus, OH) for 5 min (day 0) under bright light, to minimize stress related to a novel environment. On day 1, the rats were allowed to interact with two objects for 5 min. Sixty minutes later, the animals were re-introduced into the arena for 3 min, this time with one familiar and one novel object. The position of the objects was always the same in the arena to remove any spatial memory component from the task. Twenty-four h later (day 2), the rats were again exposed to the familiar object and another novel object for 3 min. Finally, 5 min later, the animal was exposed once more to the familiar object and another novel object for 3 min. The animals were filmed with a camera (Sony Handycam DCR-SX45) and the interaction time (nose at 2 cm or less in the object's direction) with the novel and familiar objects, and distance traveled (cm) were analyzed with the Top Scan 2.0 tracking software (Clever Systems Inc., Heston, VI, USA).

### Elevated plus maze (EPM)

On day 3, animals were placed in the center of the EPM apparatus (maze: 100 × 100 cm, arms: 10 × 45 cm) and tested for 5 min to evaluate anxiety and explorative behaviors. This task opposes rodents' innate fear of open bright spaces to their desire to explore new environments. Security was provided by the closed arms whereas the open arms offer exploratory values. Distances traveled (cm), as derived from the tracking system (Panlab), were compared.

### Open field (OF)

Like EPM, this task opposes the conflict between rodents' innate fear of the open bright center area of the arena (90 × 90 × 40 cm, black plastic) vs. their desire to explore a new environment (day 4). Distances traveled (cm) in the areas of the maze derived from the tracking system (Panlab) were compared. Security was provided by the walls of the arena.

### Morris water maze (MWM)

Following the first week of behavioral testing (NOR, EPM, OF), the animals were allowed to rest for 2 days. Then, the long-term reference memory version of the MWM, a hippocampus-dependent behavioral task, was used to investigate spatial memory. A cued test, including three trials of 60 s in which the platform was visible and positioned in the middle of the pool, was conducted on day 0 to assess age-related visual deficits and motivation to escape from water. A flag was positioned on the platform as a visual cue. On day 1, the rats were required to find in a pool (diameter: 150 cm; height: 60 cm) a submerged platform (15 cm in diameter) located 2 cm below the surface of water (24°C), rendered opaque by the addition of non-allergenic white gouache paint. Animals were pseudo-randomly started from a different position on each trial and used distal visual-spatial cues to find the hidden escape platform that remained in the middle of the same quadrant throughout training. The rats were given three trials of 90 s per day for four consecutive days (day 1–4). Animals were guided to the platform if it was not localized within 90 s. All the rats remained on the platform for 15 s before removal. Sixty minutes after the acquisition phase on day 4, the rats were given one probe trial of 90 s for which the platform was removed from the pool to assess short-term memory (learning probe). The distance traveled in each quadrant was measured using a video tracking system. At the end of the probe test, the visible platform was re-introduced into the pool to confirm motivation to escape water, visual acuity and ruled out thigmotaxis.

After 1 day of rest (day 5), all animals were submitted to a reverse memory paradigm to evaluate reversal learning. Briefly, the platform was moved to the opposite quadrant while the position of visual cues stayed the same. The rats were then trained to find the new hidden platform location for four consecutive days, as described above. A second probe test for which the platform was removed was conducted at the end of day 9 (reverse probe). Following the reversal probe trial, the visible platform was re-introduced. Finally, a third probe test was done 7 days later to reactivate long-term memory processes. Animals were sacrificed by quick decapitation 2–3 h after the last MWM probe test to allow IEG expression (day 16). After each trial, the rats were immediately placed under a heat lamp to dry and prevent hypothermia. Data derived from the MWM task were recorded on computer using a video tracking system and analyzed with the SMART 2.0 software (Panlab/Harvard Apparatus, Barcelona, Spain).

#### MWM exclusion criteria

If the performances of old rats in the cued test third trial exceeded by two times the standard deviation of the representative mean of young rats, individuals were excluded for a lack of motivation to escape water. Only one 38-month-old female LOU rat of the O/O group showed motivation to escape water (Figure 2B) therefore the entire age group was excluded. Three rats of the OCR group (3/11) also lacked motivation to escape water during the cued test and were excluded from the analysis.

Swimming speed was also used as a control for motor function, a parameter that could potentially be altered by aging. One out of three 38-month-old male LOU rats had intact motor function for swimming (average swim speed >15 cm/s) so this group was excluded. Moreover, the CHUM Research Center Animal Care Committee did not allow MWM testing on the 42-month-old female LOU rats (*n* = 3). O/O LOU rats with intact visual and motor function (7 females, 38 months old) swam every day along with other age groups. Two 20-month-old SD rats (one OAL, one OCR) did not swim properly (average swim speed <15 cm/s) and were excluded from the analysis.

### Tissue preparation and biochemical analysis

The hippocampus and adjacent cortex (entorhinal, perirhinal, and portions of adjacent neocortices) from one half-brain were isolated for immunoblotting, snap frozen and stored at −80°C. The other half-brain was fixed in 4% paraformaldehyde for 24 h, cryoprotected in 30% sucrose for 48 h, and stored at −80°C for immunochemistry. Blood was collected at sacrifice (13:00–16:00) to measure immunoreactive serum CORT concentrations.

### Corticosterone measurements

Blood was quickly centrifuged at 5000 × g for 5 min to separate and collect the serums. The CORT level was quantified for each animal in duplicate with an enzyme immunoassay (EIA, Immunodiagnostic Systems, Boldon, UK) (Veyrat-Durebex et al., 2009). Briefly, the EIA is a competitive assay utilizing a polyclonal CORT antibody, horseradish peroxidase and chromogenic substrate. The concentration of CORT was calculated from a calibration curve and samples had to be diluted by 10 with the Sample Diluent provided.

### Immunoblotting

Tissues were randomly chosen for both rat strains with equal number of male and female LOU rats (except for the very old group). Brain tissues were homogenized with a polytron in 2 ml of Tris-acetate buffer (50 mM, pH 7.4) containing 100 μM EGTA and protease inhibitors (leupeptin 5 μM, phenylmethylsulfonyl fluoride 200 μM, N-tosyl-L-phenylalanine chloromethyl ketone 1 μg/ml; Sigma-Aldrich Canada, Oakville, ON, Canada). Protein concentrations were determined using the bicinchoninic acid protein assay kit (Pierce, Rockford, IL, USA). Western blot analysis was carried out on aliquots of homogenates obtained from both aging SD and LOU rats. Ten-μg protein samples were loaded on 4–20% Tris-Glycine gels and subjected to sodium dodecyl sulfate polyacrylamide gel electrophoresis on denaturing NuPAGE® Novex 4–20% Bis-Tris gel (Invitrogen, Carlsbad, CA, USA). Proteins were transferred onto Hybond-C nitrocellulose membranes (Amersham Biosciences, Little Chalfont, UK). To block non-specific sites, membranes were first incubated for 1 h at room temperature (RT) in phosphate-buffered saline (PBS) containing 2% bovine serum albumin (BSA). Membranes were next incubated with primary antibodies directed against AMPA (GluR1 and GluR2, Abcam, Cambridge, MA, USA), NMDA (NR1, Santa Cruz Biotechnology, Santa Cruz, CA, USA; NR2A and NR2B, Abcam), group 1 mGluR (mGluR1α and mGluR5, Millipore, Billerica, MA, USA), Homer 1a (Santa Cruz Biotechnology), Arc (Cell Signaling, Danvers, MA, USA), or Zif268 proteins (Abcam) in PBS containing 2% BSA. Bands corresponding to proteins were detected with a peroxidase-conjugated secondary antibody (Santa Cruz Biotechnology) and Western Lightning Chemiluminescence Reagent Plus (Perkin Elmer, Boston, MA, USA) on Kodak BioMax MS film (Amersham Biosciences). Non-specific labeling was assessed by replacing the primary antibody with an equivalent volume of PBS-BSA. Immunoreactive levels of actin were used as a loading control. Immunoblots were placed on a Northern light illuminator and computer-generated images were analyzed semi-quantitatively by densitometry with a microcomputer imaging system (Imaging Research, MCID, St. Catharines, ON, Canada).

### Pdyn and mGluR5 immunohistochemistry (IHC)

Half-brains from both rat strains were randomly chosen for sectioning. Twenty-μm coronal sections of fixed brains at the level of the dorsal hippocampus were mounted on Superfrost Plus slides (Thermo Scientific, Portsmouth, NH, USA) and processed for IHC. Briefly, the slices were washed in PBS (pH 7.4) for 5 min then immersed in methanol containing 0.3% H_2_O_2_ for 30 min at RT to quench endogenous peroxidases. After PBS washes (3 × 5 min), sections were preincubated with 10% normal goat serum (NGS) in PBS at RT for 1 h, followed by incubation with the polyclonal mGluR5 (dilution 1/5000, Millipore) or Pdyn (dilution 1/1000, Millipore) primary antibodies (in PBS with 1% NGS) overnight at 4°C. Subsequently, sections were washed 3 times in PBS and incubated with biotinylated anti-guinea pig (Pdyn) or anti-rabbit (mGluR5) IgGs (1:1000; Vector Laboratories, Burlingame, CA, USA) in 1% NGS for 30 min at RT. After washing in PBS (4 × 10 min), sections were incubated with the avidin biotinylated enzyme complex (ABC reagent, Vector Laboratories) in PBS for 30 min. The peroxidase reaction was carried out with 0.02% H_2_O_2_ and 3,3′-diaminobenzidine tetrahydrochloride (0.1% in 100 mM Tris-HCl buffer, pH 7.4). Sections were then washed in tap water, cleaned, and coverslipped. IHC staining was visualized using bright-field microscopy (Nikon Eclipse, Melville, NY, USA) at 4 × and 20 × magnification and quantified with MCID (Imaging Research). Controls were performed using the same labeling procedure, without the primary or secondary antibodies and preadsorbtion of primary antibodies with specific peptides (data not shown).

### IEG immunofluorescence

Half-brains for both rat strains were randomly chosen for sectioning. Twenty-μm coronal sections of fixed brains at the level of the dorsal hippocampus were mounted, then washed in PBS (pH 7.4) for 5 min. Sections were permeabilized with 0.2% Tween 20 in PBS (PBST) for 10 min at RT then processed for immunofluorescence labeling. In brief, sections were incubated in 10% NGS (or normal horse serum for Homer 1a) diluted in 0.1 M PBS with 0.05% Tween 20 (PBST) and 1% BSA for 60 min at RT, followed by overnight incubation with each primary antibody (1/100 for Homer 1a and Arc, and 1/500 for the neuronal marker MAP2, Abcam) at 4°C in 0.1 M PBST containing 1% serum and BSA. After 3 washes in PBS, sections were incubated with corresponding secondary antibodies (1:500, Invitrogen, Carlsbad, CA, USA) conjugated with Alexa Fluor 488 or Alexa Fluor 568 in PBST containing 1% BSA for 2 h at RT in the dark. Sections were washed in PBS (3 × 5 min each, in the dark). Nuclei were stained with Hoechst solution (2 μg/ml, Invitrogen) for 5 min, and the sections were washed and coverslipped using fluoromount-G (Southern Biotech, Birmingham, AL, USA). Pictures were taken at 40 × magnification with an Axio Observer microscope with Apotome (Carl Zeiss MicroImaging GmbH, Jena, Germany).

### Statistics

All data are expressed as the mean ± s.e.m. ANOVA followed by Bonferroni's *post-hoc* test was used to assess the level of significance between groups for NOR, MWM, EPM, and OF parameters and protein levels. Correlation between serum CORT levels and age was analyzed using the two-tailed Pearson's test (Prism 4, GraphPad Software Inc., La Jolla, CA). Significance was established at *p* < 0.05.

Principal component analysis (PCA) was used to examine patterns of intercorrelations between the variables (Moura et al., 2010). Principal components produced by PCA are linear combinations of the original measures reflecting independent characteristics (or dimensions) underlying the correlation matrix. The first component explains most of the variance (expressed in terms of the first eigenvalue), the second component explains most of the remaining variation, and so forth. The loading of each measure on a principal component represents the correlation between the latent characteristic and the original measure and thus indicates the importance of a measure for a principal component. Measures with high loadings on the same principal component of the same sign are positively correlated, and loadings of the opposite sign are negatively correlated. Original data sets of each individual rat (*n* = 50) containing 16 variables (age, BW, NOR average, EPM open arms, OF center, serum CORT, GluR1, GluR2, NR1, NR2A, NR2B, mGluR1α, mGluR5, Homer 1a, Arc, and Zif268) were standardized using z-scores and analyzed to obtain the correlation matrix and PCA. Multivariate analyses were performed using the MATLAB software. MWM data were omitted as no reliable data could be obtained in O/O LOU rats.

## Results

### Aging marginally affects LOU rats' BW and recognition memory is intact in male and female LOU rats up to 38 and 42 months of age, respectively

**Figure 1:**
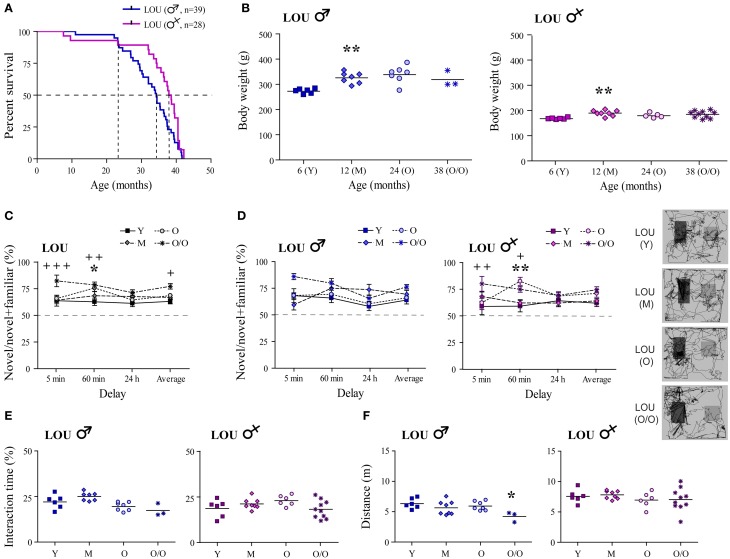
**Male and female LOU rats are characterized by high longevity, stable body weight, and intact recognition memory. (A)** Median survival age was 38.2 months for females vs. 34.3 months for males LOU rats. **(B)** BW of male and female LOU rats increased only slightly in the course of aging (Y, young adult; M, mature; O, old; O/O, old/old). **(C)** Amount of time spent interacting with novel objects did not decrease with age in LOU rats, and O/O rats spent significantly more time with novel objects on average and after the 5- and 60-min delay than young rats. **(D)** Recognition memory is intact in males and females LOU rats up to 38 and 42 months of age, respectively, with significant differences only for females. Representative paths (60 min delay) are shown with novel object on the right side. **(E)** Average interacting time with the objects was similar at different ages. **(F)** Distance traveled in the arena was not different between LOU age groups for females but lower for O/O males. Values are means ± s.e.m.; *n* = 12–15/age group. Significance of changes was determined by One-Way ANOVA (Two-Way for NOR), followed by Bonferroni post-test, Y vs. O: ^*^p < 0.05, ^**^p < 0.01; Y vs. O/O: ^+^*p* < 0.05, ^++^*p* < 0.01, ^+++^*p* < 0.001.

Increased longevity has been reported for the LOU rat strain (Alliot et al., 2002). In our study, median survival age was evaluated at 38.2 months for females vs. 34.3 months for males LOU rats (Figure 1A, curves comparison: *p* = 0.0181). To evaluate survivability, only natural deaths were considered (*n* = 28–39/group); sacrifices for experimentation and deaths related to surgeries were excluded. Survival proportions were similar between males and females groups up to 24 months of age. Then, the male population slowly decline while that of females remained stable up to 32 months of age. Aging is generally associated with a large BW gain in rodents. In male LOU rats, a 19% increase was observed between 6- and 12 months but BW was stable thereafter, even at a very advanced age (Figure 1B, *n* = 3–7). In females, BW changes were even slighter (Figure 1B, *n* = 5–10).

Intact recognition memory has previously been observed in 24-month-old male LOU rats (Kollen et al., 2010). Here, we report for the first time that up to 38–42 months, male and female LOU rats spent significantly more time with novel objects at every delay tested (Figure 1C, *n* = 12–15), suggesting the maintenance of an intact recognition memory in this strain at advanced age. O/O rats interact even more with the novel objects than young rats [age effect Y vs. O/O: Two-Way ANOVA, *F*_(1, 69)_ = 17.77, *p* = 0.0003, *n* = 12–13]. Time delays of novel object exposure affected old and very old male LOU rats with higher percentage of time interacting with the novel object after the 5-min delay [Figure 1D, delay effect Y vs. O: Two-Way ANOVA, *F*_(3, 33)_ = 4.12, *p* = 0.0138, *n* = 6–7; Y vs. O/O: *F*_(3, 21)_ = 6.09, *p* = 0.0039, *n* = 3–6]. No significant difference was observed for aging female LOU rats. Total interaction times with the objects remained similar at all ages in male and female LOU rats (Figure 1E). Aging had no effect on motor function in female LOU rats however total distance traveled in the NOR arena was reduced by 33% in very old males compared to young animals [Figure 1F; One-Way ANOVA, *F*_(3, 20)_ = 3.601, *p* = 0.0315, *n* = 3–6].

### Spatial memory is intact in 24-month-old male and female LOU rats

**Figure 2:**
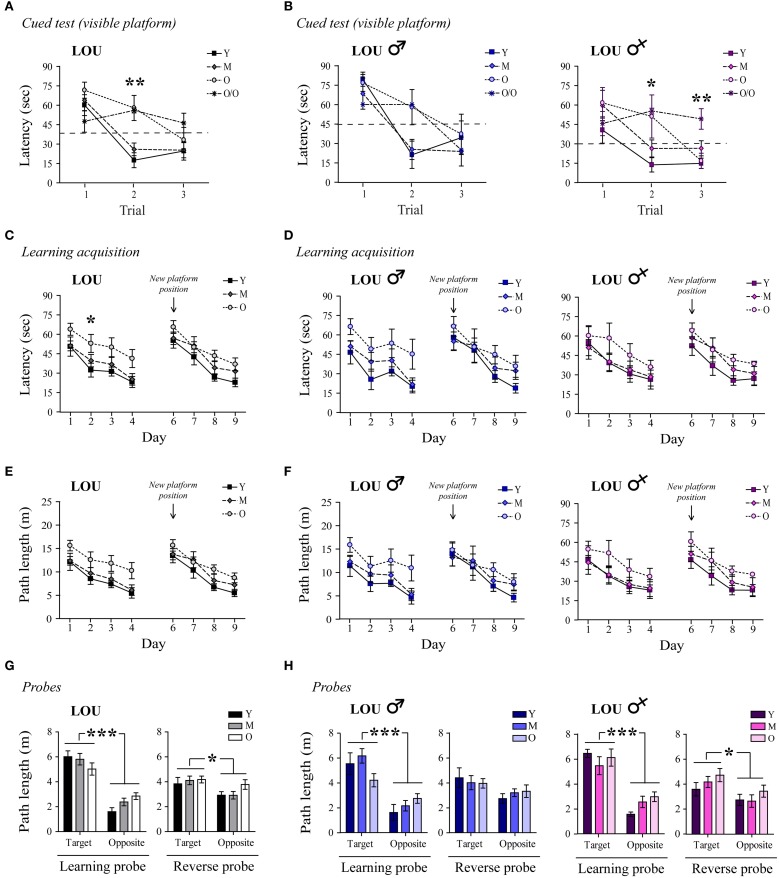
**Spatial memory acquisition is intact in old LOU rats. (A)** All animals except the O/O LOU rats learned to swim to the visible platform to escape the water after three trials of 60 s (delay of 30 min between each trial, see Materials and Methods, for exclusion criteria). A flag was positioned on the platform as a visual cue. **(B)** Very old female LOU rats lacked motivation to escape water. **(C)** Morris Water Maze (MWM) learning acquisition curves of aging LOU rats (day 1–4, three trials per day). Old LOU rats took more time to find the platform only on day 2. After 1 day of rest, the platform was moved to the opposite quadrant to assess inhibitory learning acquisition (day 6–9, three trials per day). Performances were similar for all LOU rats despite aging. **(D)** No significant difference was observed when individual performances were analyzed by gender. **(E,F)** To rule out an effect of motor function, path lengths to reach the platform were compared. Again, performances were similar for all LOU rats. **(G)** Aging did not affect LOU rat performances in the retention probe tests conducted 60 min after the last trial on day 4 (learning) and 9 (reverse), as distance swum in target quadrants was similar for all groups but significantly higher when compared to the opposite quadrant. **(H)** Female LOU rats performed better in the reverse probe test. Values are means ± s.e.m.; *n* = 12–15/age group. Significance of changes for latencies and path length was determined by Two-Way ANOVA followed by Bonferroni post-test, ^*^p < 0.05, ^**^p < 0.01, ^***^p < 0.001.

We next examined whether or not hippocampus-dependent spatial memory was preserved in aging male and female LOU rats, using the MWM reference task. First, a cued test for which the platform was visible was conducted to assess swimming ability, motivation, visual, and motor functions. O/O LOU rats lack motivation to escape water and did not improve their performance during the cued test (Figure 2A, trial 1: 47.5 s ± 8.3 s, trial 3: 46.3 s ± 7.6 s, *n* = 8–15), therefore this group was excluded from the analysis (see Materials and Methods, MWM Exclusion Criteria section). Conversely, LOU rats in other age groups learned to escape the pool after three trials [time effect: Two-Way ANOVA, *F*_(2, 72)_ = 26.01, *p* < 0.0001, *n* = 12–15].

Likewise, old LOU rats appeared to take more time to acquire the task and find the hidden platform (day 1–4) compared to young LOU rats [Figure 2C, age effect: Two-Way ANOVA, *F*_(2, 108)_ = 8.05, *p* = 0.0013, *n* = 12–15]. Nevertheless, old LOU rat latencies consistently decreased as in young animals, suggesting slower learning speed but efficient spatial memory (linear regression young: slope = −8.31, *R*^2^ = 0.8642; old: slope = −7.05, *R*^2^ = 0.9590, *n* = 12–15). Our group and others have shown that reversal learning and memory is affected by aging. Old mice, rats, and monkeys cannot discriminate between two related tasks as well as adult animals (Burke et al., 2011; Menard and Quirion, 2012b; Menard et al., 2013). To evaluate reverse spatial memory function, the platform was moved to the opposite quadrant of the pool on day 6 and learning was studied for four consecutive days (day 6–9). For this task, no significant difference was observed between the LOU rat age groups, suggesting intact reversal learning acquisition despite aging (Figure 2C). We also examined if gender differentially affects spatial learning acquisition in old LOU rats. As shown on Figure 2D, learning acquisition was slower for 24-month-old male LOU rats [age effect: Two-Way ANOVA, *F*_(2, 51)_ = 5.50, *p* = 0.0144, *n* = 6–7] but no significant difference was observed for reversal learning [age effect: Two-Way ANOVA, *F*_(2, 51)_ = 0.94, *p* = 0.4108, *n* = 6–7]. In contrast, old female LOU rats successfully learned both tasks. Swim speed may decline with age thus we compared path lengths to reach the platform on training trials (Figure 2E). We obtained similar results with an effect of aging only for the learning acquisition task (day 1–4) of old male LOU rats [Figure 2F, Two-Way ANOVA, *F*_(2, 51)_ = 4.82, *p* = 0.0219, *n* = 6–7]. Finally, memory retention was measured by conducting probe trials with the platform removed 60 min after the last hidden platform training on day 4 of learning and day 9 of reverse acquisition tasks. LOU rats favored the target quadrant in both probe tests [Figure 2G, Two-Way ANOVA learning quadrant: *F*_(1, 36)_ = 72.23, *p* < 0.0001; reverse quadrant: *F*_(1, 36)_ = 5.74, *p* = 0.0219, *n* = 12–15], confirming efficient spatial memory. While both male and female LOU rats favored the target quadrant in the learning probe test [Figure 2H, Two-Way ANOVA quadrant effect males: *F*_(1, 17)_ = 30.42, *p* < 0.0001; females: *F*_(1, 16)_ = 44.02, *p* < 0.0001], the difference was significant only for females during the reverse probe trial [males: *F*_(1, 17)_ = 4.03, *p* = 0.0608; females: *F*_(1, 16)_ = 5.60, *p* = 0.031].

### LTCR prevents age-related BW gain and recognition memory deficits in SD rats

**Figure 3:**
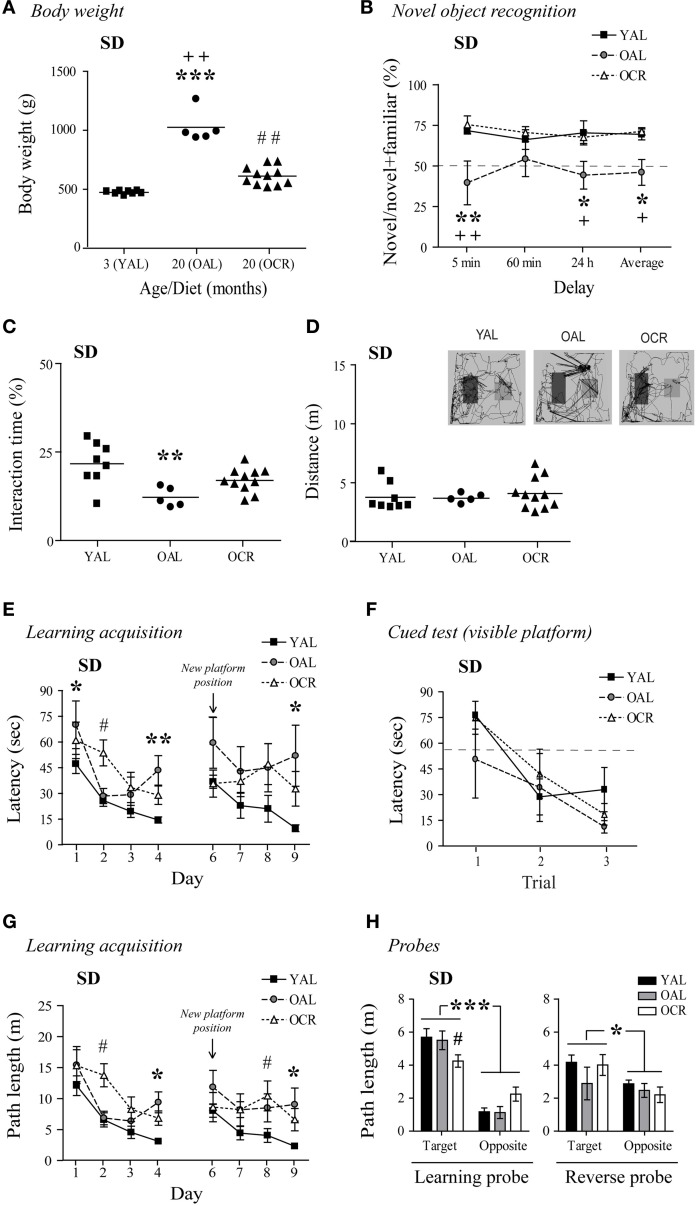
**Long-term caloric restriction prevents age-related body weight increase and minimizes the cognitive deficits observed in old AL male SD rats**. **(A)** The age-related increase in BW is prevented by LTCR in SD rats (YAL, young *ad-libitum*-fed; OAL, old *ad-libitum*-fed; OCR, old calorie-restricted). **(B)** OAL SD rats spent less time interacting with novel objects on average and after a 5-min or 24-h delay compared to YAL and OCR rats. **(C)** OAL SD rats spent less time interacting with the objects during the test compared to YAL rats. **(D)** Distance traveled in the arena was not different between SD age groups. Representative paths (60 min delay) are shown with novel object on the right side. **(E)** MWM learning acquisition curves of the YAL, OAL, and OCR SD rats (three trials per day). Performances were significantly different when compared to YAL for OAL on day 1, 4, and 9, and OCR on day 2. These differences were not related to visual deficits, motor function, or lack of motivation to escape water as assessed by the cued test **(F)**. **(G)** Longer path lengths on the last day of both learning (day 4) and reversal learning (day 9) acquisition confirmed memory deficits in the OAL group. **(H)** Path length in the target quadrant was lower in OCR for the learning probe test (conducted 60 min after the last trial on day 4), while no difference was seen for the reverse probe test (60 min after the last trial on day 9). Path lengths were also significantly higher when compared to the opposite quadrant. Values are means ± s.e.m.; *n* = 5–11/age group (*n* = 4–8/age group for MWM, see Materials and Methods, for exclusion criteria). Significance of changes in BW, total interaction times, and distances traveled were determined by One-Way ANOVA followed by Bonferroni post-test. Significance of changes in percentages of time spent with novel objects, latencies, and path lengths were determined by Two-Way ANOVA followed by Bonferroni post-test, YAL vs. OAL: ^*^p < 0.05, ^**^p < 0.01, ^***^p < 0.001; YAL vs. OCR: ^#^*p* < 0.05, ^##^*p* < 0.01; OAL vs. OCR: ^+^*p* < 0.05, ^++^*p* < 0.01.

Cognitive functions generally decline with aging in humans and metabolic disturbances related to diabetes and obesity can exacerbate these deficits. In fact, diabetes has been associated with declining cognitive performance in aging (Umegaki et al., 2013), and elevated levels of circulating glucose and insulin, as found in OAL SD rats, are prevented by LTCR (Bedard et al., 2010). In AL male SD rats, aging was associated with a large BW gain as previously reported (Bedard et al., 2010, 2013; Moyse et al., 2012). In 20-month-old AL rats, BW was 216% higher than in YAL rats, and LTCR prevented this increase [Figure 3A, One-Way ANOVA, *F*_(2, 21)_ = 73.49, *p* < 0.0001, *n* = 5–11].

In the NOR task, 20-month-old AL SD rats spent 32, 12, and 26% less time with the novel objects than YAL rats after 5-min, 60-min, and 24-h delays, respectively (Figure 3B). Interestingly, LTCR prevented this decline [diet effect: Two-Way ANOVA, *F*_(1, 42)_ = 16.55, *p* = 0.0012, *n* = 5–11]. OAL SD rats' total interaction time with the objects decreased by 5–10% compared to YAL and OCR rats [Figure 3C, One-Way ANOVA, *F*_(2, 21)_ = 7.343, *p* = 0.0038, *n* = 5–11]. The decreases observed in OAL SD rats were not related to lower exploratory behavior, since no difference was observed in the total distance traveled in the NOR arena between age groups [Figure 3D; One-Way ANOVA, *F*_(2, 21)_ = 0.2844, *p* = 0.7553, *n* = 5–11].

We next evaluated if aging and diet affects spatial memory in this rat strain using the MWM task. In the cued test, all SD rats learned to escape the pool after three trials [Figure 3F, time effect: Two-Way ANOVA, *F*_(2, 32)_ = 10.39, *p* = 0.0003, *n* = 4–8]. As shown in Figure 3E, YAL SD rats quickly learned to find the hidden platform (day 1–4). However, aging affected OAL rat performances, as this group exhibit longer escape latencies than younger rats on day 4 [One-Way ANOVA, *F*_(2, 16)_ = 8.226, *p* = 0.0035, *n* = 4–8]. The learning acquisition curve was significantly different for the OAL group compared to young animals [age effect: Two-Way ANOVA, *F*_(1, 30)_ = 12.23, *p* = 0.0058, *n* = 4–8], and LTCR appeared ineffective to improve spatial memory in old SD rats [diet effect: Two-Way ANOVA, *F*_(1, 27)_ = 0.03, *p* = 0.8641, *n* = 4–7]. However, a comparison of curve slopes revealed similar learning patterns for young and OCR rats (linear regression YAL: slope = −10.53, *R*^2^ = 0.8787; OCR: slope = −11.69, *R*^2^ = 0.9402, *n* = 7–8) while OAL latencies increased on day 4, affecting learning progress (linear regression OAL: slope = −10.15, *R*^2^ = 0.3241, *n* = 4). In the reverse learning task, YAL SD rats quickly swam to the new hidden platform position (Figure 3E). Conversely, both OAL and OCR rats performed poorly [YAL vs. OAL: Two-Way ANOVA, *F*_(1, 30)_ = 6.04, *p* = 0.0338; YAL vs. OCR: Two-Way ANOVA, *F*_(1, 39)_ = 5.82, *p* = 0.0314, *n* = 4–8]. Comparison of curve slopes and coefficient of determination confirmed reverse memory deficits in old SD rats (linear regression YAL: slope = −8.42, *R*^2^ = 0.9397; OAL: slope = −2.05, *R*^2^ = 0.1199; OCR: slope = 0.09, *R*^2^ = 0.0004, *n* = 4–10). Nonetheless, only OAL rats took significantly more time to escape the pool on day 9 in comparison to young animals [Figure 3E, One-Way ANOVA, *F*_(2, 16)_ = 4.923, *p* = 0.0216, *n* = 4–8]. Path lengths to reach the platform on training trials confirmed memory impairments for OAL rats with significant differences on day 4 [Figure 3G, One-Way ANOVA, *F*_(2, 16)_ = 9.151, *p* = 0.0022, *n* = 4–8] and 9 [One-Way ANOVA, *F*_(2, 16)_ = 4.762, *p* = 0.0238, *n* = 4–8]. In comparison to young animals, path length in the target quadrant was significantly lower for OCR rats in the learning probe test but distance traveled in the target quadrant was significantly higher for all SD rat groups when compared to the opposite quadrant [Figure 3H, Two-Way ANOVA learning quadrant: *F*_(1, 16)_ = 68.87, *p* < 0.0001], indicating intact memory retention. No significant difference was seen in the reverse probe test. Overall these results suggest that obese 20-month-old SD rat recognition memory is impaired and spatial memory acquisition less efficient in comparison to young animals. Interestingly, LTCR partially prevented these deficits.

### Anxiety is low in aging LOU rats and reduced in old CR SD rats

**Figure 4:**
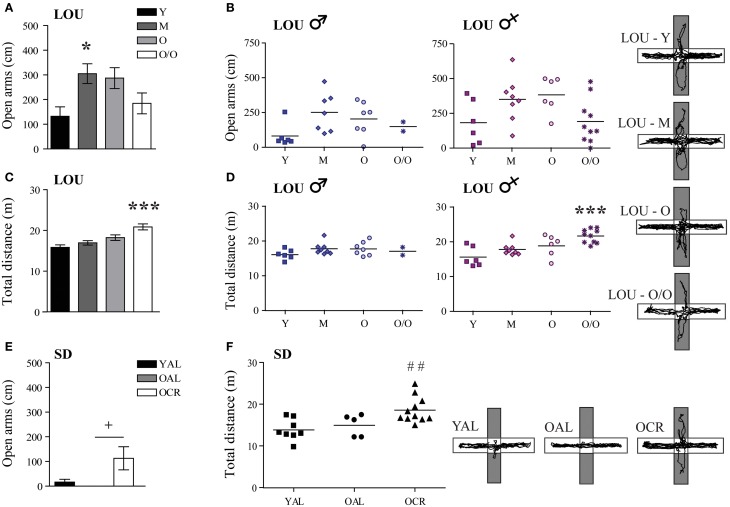
**LOU rats exhibit a low anxiety phenotype in the elevated plus maze task and LTCR reduces anxious behavior in old SD rats**. **(A)** LOU rats traveled over 100 cm on the EPM open arms, revealing high explorative behaviors and low anxiety. **(B)** No significant difference was observed between aging male and female LOU rats. **(C)** Locomotor activity increased with age for the LOU rats to become significantly different in O/O rats. **(D)** This effect is specific to females. **(E)** OAL SD rats did not explore the EPM open arms, indicating stress-related anxious behaviors. LTCR enhanced explorative behaviors in old SD rats. **(F)** OCR SD rats traveled a higher total distance in the EPM arena compared to YAL rats, suggesting lower anxiety and higher explorative behavior in a bright environment. Representative paths are shown (open arms in gray). Values are means ± s.e.m.; *n SD* = 5–11/age group; *n LOU* = 12–15/age group. Significance of changes for distances traveled in the open arms and total distances were determined by One-Way ANOVA followed by Bonferroni post-test, ^*^p < 0.05, ^***^p < 0.001; YAL vs. OCR: ^##^*p* < 0.01; OAL vs. OCR: ^+^*p* < 0.05.

Normal aging is associated with increased anxiety and cognitive decline in mice (Menard et al., 2013) and humans (Bunce et al., 2012). In adult mice, caloric restriction reduces anxiety-related behaviors (Yamamoto et al., 2009). Therefore, we studied stress responses and explorative behavior in both aging rat models, using the EPM and OF tasks. In EPM, anxiety-like behaviors and exploration were measured by the distance traveled in the open arms of the maze. In the LOU rat strain, mature animals exhibited the highest exploratory behavior in the EPM open arms [Figure 4A, One-Way ANOVA, *F*_(3, 48)_ = 4.061, *p* = 0.0119] and aging did not increase their levels of anxiety (Y: 132.33 ± 38.27 cm, O/O: 184.64 ± 42.16 cm, *n* = 12–15). Male and female aging LOU rat exploratory activity was similar in this task (Figure 4B). Locomotor activity gradually increased with age to become significantly different for O/O LOU rats compared to other age groups [Figure 4C, One-Way ANOVA, *F*_(3, 48)_ = 10.66, *p* < 0.0001, *n* = 12–15]. This difference was associated with the female group (Figure 4D). In contrast, none of the AL 20-month-old SD rats explored the EPM open arms (Figure 4E, YAL: 16.50 ± 11.22 cm, OAL: 0 ± 0 cm, *n* = 5–8) despite intact locomotor activity (Figure 4F, YAL: 1388.2 ± 89.72 cm, OAL: 1499.05 ± 117.48 cm, *n* = 5–8). Interestingly, caloric restriction significantly enhanced both exploratory behavior [Figure 4E, One-Way ANOVA, *F*_(2, 21)_ = 3.867, *p* = 0.0390, 112.91 ± 46.68 cm, *n* = 5–11] and locomotor activity [Figure 4F, One-Way ANOVA, *F*_(2, 21)_ = 7.163, *p* = 0.0042, 1860.75 ± 92.58 cm, *n* = 5–11].

**Figure 5:**
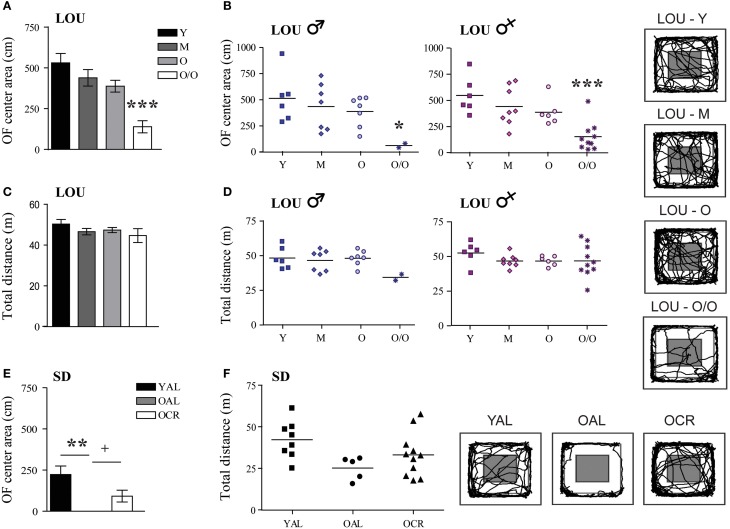
**Decreased anxiety-related behavior is observed in old LOU and CR SD rats in the open field task. (A)** In contrast to EPM results, O/O LOU rat exploratory behavior in the OF center area of the arena was significantly reduced. **(B)** This difference was observed in both O/O males and females. Representative paths are shown (center area in gray). Neither aging **(C)** nor gender **(D)** affected total distances traveled. **(E)** OAL SD rats did not explore the center area, suggesting high anxiety as in the EPM test. LTCR prevented the development of anxious behavior as observed in OCR SD rats. **(F)** The total distance traveled was not significantly different between the three groups. Representative paths are shown. Values are means ± s.e.m.; *n SD* = 5–11/group; *n LOU* = 12–15/group. Significance of changes in distances traveled in the OF center area and total distances were determined by One-Way ANOVA followed by Bonferroni post-test, ^*^p < 0.05, ^**^p < 0.01, ^***^p < 0.001; OAL vs. OCR: ^+^*p* < 0.05.

In contrast to EPM results, the distance traveled in the center area of the OF arena decreased with aging in LOU rats [Figure 5A, One-Way ANOVA, *F*_(3, 48)_ = 12.03, *p* < 0.0001, Y: 531.00 ± 57.21 cm, M: 438.92 ± 50.71 cm, O: 387.79 ± 36.31 cm, O/O: 138.91 ± 37.29 cm, *n* = 12–15]. Both male and female O/O LOU rats displayed anxious behaviors (Figure 5B) although their locomotor activity remained stable [Figure 5C, One-Way ANOVA, *F*_(3, 48)_ = 1.075, *p* = 0.3685, *n* = 12–15] with no gender difference (Figure 5D). Nevertheless, in comparison to OAL SD rats, mean distances traveled in the center area were significantly higher for LOU rats up to 42 months of age [One-Way ANOVA, *F*_(4, 52)_ = 16.01, *p* < 0.0001, *n* = 5–15]. In fact, none of the OAL SD rats reached the center of the arena, suggesting a high anxiety state [Figure 5E, One-Way ANOVA, *F*_(2, 21)_ = 5.922, *p* = 0.0091, YAL: 222.78 ± 51.87 cm, OAL: 0 ± 0 cm, OCR: 91.68 ± 35.91 cm, *n* = 5–11]. Locomotor activity did not change significantly between SD groups [Figure 5F, One-Way ANOVA, *F*_(2, 21)_ = 2.949, *p* = 0.0743, *n* = 5–11]. Our results suggest that LTCR reduces anxiety-related behaviors associated with aging and that the obesity-resistant LOU rat strain could be considered as a low-anxiety phenotype.

### Circulating corticosterone levels are stable in mature to very old LOU rats and increased in aging SD rats while prodynorphin is overexpressed in the hippocampus and entorhinal cortex of old AL SD rats

**Figure 6:**
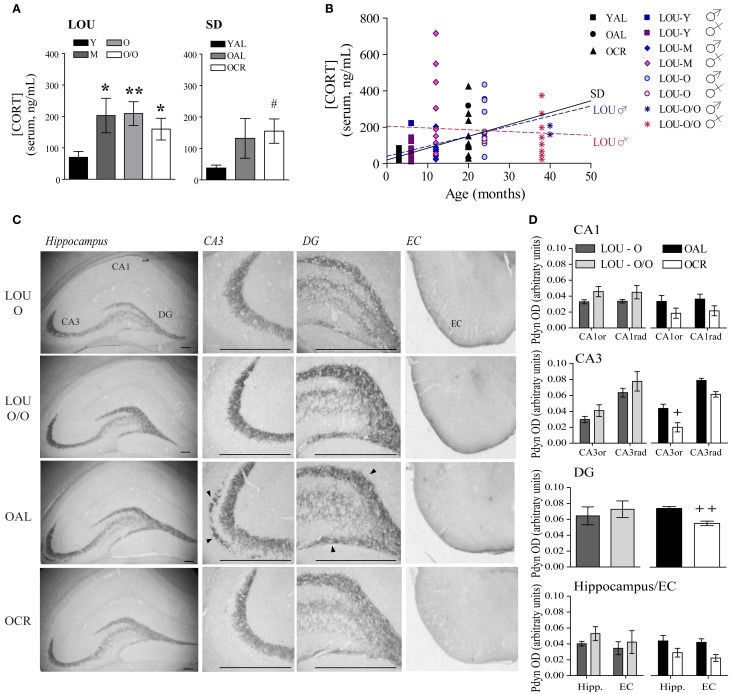
**The endogenous opioid prodynorphin is overexpressed in OAL SD rat hippocampus and entorhinal cortex. (A)** Enhancement of circulating CORT was observed in 12-month-old LOU rats but remained stable thereafter. CORT levels increased in old SD rats and were significantly correlated with age in male SD and male LOU rats **(B)**. **(C)** Pdyn immunostaining is localized in the CA3 and DG hippocampal areas and entorhinal cortex. Scale bar at 500 μm. Arrowheads indicate Pdyn-positive cells. **(D)** While Pdyn is highly expressed in the hippocampus CA3 and DG of OAL SD rats in comparison to OCR animals, no difference was observed between O and O/O LOU rats. Values are means ± s.e.m.; *n* = 15 slices from three animals/group. Significance of changes in CORT levels was determined by One-Way ANOVA in each strain. Association of CORT levels with age was performed was assessed using Pearson's correlation. Changes in the intensity of immunostaining were determined using *t*-test for DG and Two-Way ANOVA followed by Bonferroni post-test for other brain regions, ^*^p < 0.05, ^**^p < 0.01; OAL vs. OCR: ^+^*p* < 0.05, ^++^*p* < 0.01.

Stressful events inducing the release of glucocorticoids (GC) have been associated with memory impairments in older adults (Lupien et al., 2005). Exaggerated anxiety responses, as observed in OAL SD rats, may affect memory formation and consolidation by promoting GC production. Notably, plasma CORT levels have been correlated with cognitive ability in young mice (Harrison et al., 2009). As shown in Figure 6A, CORT level was increased in 12-months-old LOU rats compared to young animals; however, it remained stable thereafter up to 38 months [Figure 6A, age effect: One-Way ANOVA, *F*_(3, 44)_ = 2.354, *p* = 0.0850, *n* = 10–15]. Serum CORT was elevated in old SD rats with a significant difference for OCR SD rats [One-Way ANOVA, *F*_(2, 18)_ = 2.823, *p* = 0.0858, *n* = 4–10; YAL vs. OCR: *p* < 0.05, *n* = 7–10] consistent with previous findings in naive OCR rats (Bedard et al., 2010). We then examined the correlation between age and circulating CORT in each strain. While serum CORT significantly increased in 20-month-old SD rats in comparison to 3-month-old animals (Figure 6B, *p* = 0.0268, *R*^2^ = 0.2327), no correlation was observed for young and aging LOU rats (*p* = 0.3329, *R*^2^ = 0.0203). Interestingly, the correlation between CORT levels and age was significant for males (*p* = 0.0154, *R*^2^ = 0.2489) but not females (*p* = 0.7224, *R*^2^ = 0.0056) LOU rats.

The endogenous opioid peptides dynorphins are released during stress response and enhance anxiety-related behaviors (Wittmann et al., 2009; Bilkei-Gorzo et al., 2012). Since *Pdyn* gene expression generally increases in normal aging (Kotz et al., 2004) and has been associated with age-related cognitive deficits (Nguyen et al., 2005; Menard et al., 2013), we examined the effect of LTCR on Pdyn levels in old SD brain. Strong Pdyn immunostaining was observed in the hippocampal CA3 region and dentate gyrus (DG) regions, as previously reported (Schwarzer, 2009) (Figure 6C). No significant difference was observed for LOU rats despite aging (Figure 6D, Two-Way ANOVA, O vs. O/O: CA1: *p* = 0.1978; CA3: *p* = 0.3085; DG: *p* = 0.6176; hippocampus/EC: *p* = 0.4641). However, quantification of immunostaining revealed a significant effect of LTCR on Pdyn expression in CA3 neurons [Figure 6D, OAL vs. OCR: Two-Way ANOVA, *F*_(1, 4)_ = 13.72, *p* = 0.0208, *n* = 15 slices from 3 rats/group] and DG (two-tailed *t*-test, *p* = 0.0067, *n* = 15 slices from 3 rats/group), in line with reduced anxiety-related behaviors. In comparison to OCR animals, Pdyn immunostaining was significantly higher in OAL hippocampus and EC [Figure 6D, Two-Way ANOVA, *F*_(1, 4)_ = 24.50, *p* = 0.0078, *n* = 15 slices from 3 rats/group]. Comparison of Pdyn expression between LOU and OCR rats revealed no significant difference between O, O/O LOU, and OCR rats [Two-Way ANOVA, CA1: *p* = 0.0554; CA3: *p* = 0.1483; DG: *p* = 0.2755; hippocampus/EC: *p* = 0.1929]. The hippocampal CA3 region is essential for encoding novelty and pattern separation (Kesner, 2007) and glutamate receptor function in this area was recently tied to individual differences in cognitive aging (Yang et al., 2013). In contrast, EC has been closely associated with working memory (Traissard et al., 2007; Newmark et al., 2013). Higher Pdyn expression in these brain areas could reduce glutamatergic transmission (Wagner et al., 1993) and impaired synaptic plasticity underlying memory processes in the OAL SD rat brain.

### Glutamate receptor levels remain unchanged in aging LOU rats and are increased in old CR SD rats

**Figure 7:**
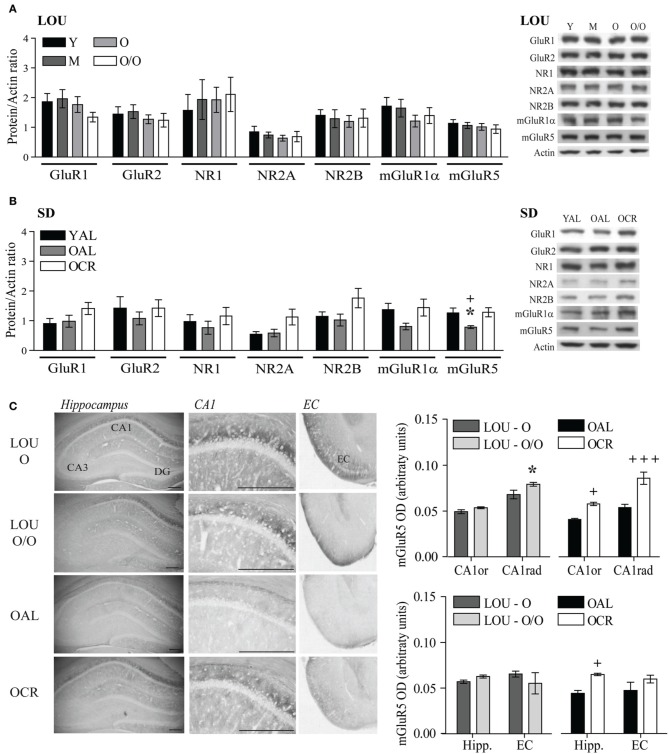
**Glutamate receptor levels are unaltered in aging LOU rats and mGluR5 expression is increased by LTCR in the hippocampus of old SD rats**. **(A)** No change for ionotropic AMPA GluR1, GluR2, NMDA NR1, NR2A, NR2B, and metabotropic mGluR1α and mGluR5 protein level was observed in LOU rat hippocampal formation up to 38–42 months of age. **(B)** mGluR5 receptor level was significantly reduced in OAL compared to young and OCR SD rats. **(C)** Quantification of immunostaining revealed higher mGluR5 expression in CA1 and overall hippocampus of OCR rats compared to OAL and a slight increase in the CA1 *stratum radiatum* (CA1rad) of O/O LOU rats. Western blot values are means ± s.e.m. of eight individual experiments and data are expressed as protein/actin ratio. Representative blots of glutamate receptor subunits and actin loading control are shown for each rat group. *n LOU* = 8/age group, *n SD* = 4–8/age group. For immunohistochemistry, values are means ± s.e.m.; *n* = 15 slices from three animals/group. Scale bar at 500 μm. Significance of changes was determined by Two-Way ANOVA followed by Bonferroni post-test, ^*^p < 0.05; OAL vs. OCR: ^+^*p* < 0.05, ^+++^*p* < 0.001.

Therefore, we assessed AMPA and NMDA receptor subunit levels in the hippocampus and adjacent cortices of aging LOU and SD rats. Tissue preparation included the whole hippocampus and adjacent cortices, since recognition memory has been related to the perirhinal cortex (Burke et al., 2011). Moreover, behavioral training was performed on a 4-week schedule, which implies consolidation of memories in the cortices (Frankland and Bontempi, 2005). Glutamate receptor protein levels were unaffected in the aging obesity-resistant LOU rat strain (Figure 7A). Moreover, no significant variation was observed between males and females (data not shown). In contrast, NMDA receptor subunit levels increased significantly in the brains of OCR SD rats compared to age-matched OAL rats [Figure 7B, Two-Way ANOVA, *F*_(1, 30)_ = 4.66, *p* = 0.0390, *n* = 4–8]. Metabotropic group 1 mGluRs play a crucial role in memory processes in the aging brain (Xu et al., 2009; Menard and Quirion, 2012a; Menard et al., 2013) and like ionotropic glutamate receptors mGluR1α and mGluR5 protein levels were maintained in the brain of aging LOU rats (Figure 7A). In contrast, protein levels of mGluR5 were significantly lower in the brain of OAL compared to OCR SD rats [Figure 7B, Two-Way ANOVA, *F*_(1, 20)_ = 5.75, *p* = 0.0263, *n* = 4–8] and young rats [Two-Way ANOVA, *F*_(1, 20)_ = 6.69, *p* = 0.0177, *n* = 4–8]. Moreover, higher protein levels were observed overall in old LOU rats compared to OAL SD rats [Two-Way ANOVA, *F*_(1, 60)_ = 5.28, *p* = 0.0444, *n* = 4–8] while the difference was not significant between old LOU and OCR SD rats [Two-Way ANOVA, *F*_(1, 84)_ = 0.09, *p* = 0.7629, *n* = 8], indicating that LTCR may insure memory protection by preventing age-related decrease of glutamate receptor expression. We investigated further expression pattern of glutamatergic synaptic markers independently between young LOU vs. SD rats. We obtained a significant difference for GluR1 (*p* < 0.05) when we analyzed AMPA receptor expression. Protein levels of NMDA NR2 subunits were also significantly increased in young LOU rats when compared to young SD rats (*p* = 0.0392).

mGluR5 receptors are enriched in the CA1 region of the hippocampus, which is crucial for working and spatial memory processing. Using immunohistochemistry, we compared mGluR5 staining in old LOU and SD rats. As shown in Figure 7C, mGluR5 immunoreactivity revealed intensely stained pyramidal neurons in the hippocampal CA1 region. mGluR5 staining was slightly increased in the CA1 *stratum radiatum* of O/O LOU rats compared to O LOU rats [CA1, O vs. O/O, Two-Way ANOVA, *F*_(1, 4)_ = 4.64, *p* = 0.0976, *n* = 15 slices/group from 3 animals]. Immunoreactivity was significantly higher in the CA1 pyramidal neurons of OCR in comparison to those of OAL SD rats [OAL vs. OCR, Two-Way ANOVA, *F*_(1, 4)_ = 26.00, *p* = 0.0070, *n* = 15 slices/group from 3 animals]. Finally, mGluR5 immunostaining was significantly enriched in the whole hippocampus and entorhinal cortex of OCR SD rats [Figure 7C, OAL vs. OCR, Two-Way ANOVA, *F*_(1, 4)_ = 20.99, *p* = 0.0102, *n* = 15 slices/group from 3 animals], while no difference was observed for LOU rats (Figure 7C).

### Homer 1a and arc immediate early genes are involved in the maintenance of memory functions in aging LOU rats and old CR SD rats

**Figure 8:**
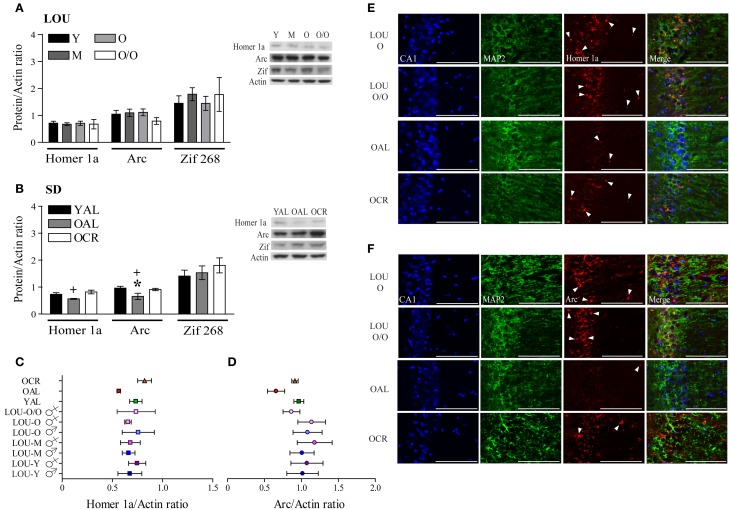
**Immediate early genes *Homer 1a* and *Arc* protein levels are maintained in the hippocampal formation of O and O/O LOU and OCR SD rats**. **(A)** In line with intact cognition and youthful levels of glutamate receptors, Homer 1a, Arc, and Zif 268 expression remained unchanged in aging LOU rats after MWM reactivation. **(B)** Conversely, both Homer 1a and Arc expression was decreased in old AL memory-impaired SD rats, in comparison to OCR rats which are characterized by intact recognition memory and spatial learning acquisition. Representative blots of IEG protein levels and actin loading control are shown for each rat group. Comparison of IEG protein levels between all rat groups is shown for Homer 1a **(C)** and Arc **(D)**. **(E)** Homer 1a co-immunolabeling with the neuronal marker microtubule-associated protein 2 (MAP2) confirmed higher expression of this IEG in aging LOU and OCR SD rats pyramidal cells compared to OAL SD rats. **(F)** Arc staining was also enhanced in the CA1 area of these rats compared to that of OAL SD rats. Western blot values are means ± s.e.m. of eight separate experiments and data are expressed as ratio of protein/actin levels. *n LOU* = 8/age group; *n SD* = 4–8/age group. Scale bar at 100 μm. Arrowheads indicate IEG-positive cells. Significance of changes for IEG protein levels were determined by Two-Way ANOVA followed by Bonferroni post-test, YAL vs. OAL: ^*^p < 0.05; OAL vs. OCR: ^+^*p* < 0.05.

Activation of glutamate receptors following behavioral training should lead to expression of IEG necessary to consolidate memories (Inoue et al., 2009; Czerniawski et al., 2011). In fact, down-regulation of IEG expression has been associated with memory impairments in aged rats (Rowe et al., 2007; Benoit et al., 2011; Penner et al., 2011; Menard and Quirion, 2012b). Scaffolding protein Homer 1a regulates group 1 mGluR activation and reduced levels have been associated with memory impairments (Menard and Quirion, 2012b; Kaja et al., 2013). On the other hand, IEG *Arc* is considered a master regulator of synaptic plasticity (Shepherd and Bear, 2011). Along with youthful levels of glutamate receptor and intact cognitive abilities, Homer 1a and Arc protein levels were unaltered in the hippocampal formation and adjacent cortices of aging LOU rats (Figure 8A). No significant variation was observed between males and females (data not shown). In line with cognitive deficits, both Homer 1a and Arc levels were significantly lower in the brains of OAL SD rats when compared to young animals [Figure 8B, YAL vs. OAL, Two-Way ANOVA, *F*_(1, 20)_ = 9.63, *p* = 0.0056, *n* = 4–8] and LTCR prevented this change [Figure 8B, OAL vs. OCR, Two-Way ANOVA, *F*_(1, 20)_ = 13.01, *p* = 0.0018, *n* = 4–8]. Zif 268, another IEG involved in the early steps of memory consolidation (Alberini, 2009), was unaltered in aging LOU and SD rats (Figures 8A,B). Homer 1a (Figure 8C) and Arc (Figure 8D) protein levels are similar in all rat groups except for the OAL SD rats. An age-related decrease of *Arc* gene transcription has been reported in the pyramidal cells of the hippocampus CA1 region of memory-impaired Fischer-344 rats (Penner et al., 2011). Since glutamate receptors mGluR5 are enriched in this area (Figure 7C) alterations of *Homer 1a* expression following behavioral training could be expected as well. Co-localization of Homer 1a with the neuronal marker microtubule-associated protein 2 (MAP2) revealed more positive cells in the CA1 region of old and very old LOU and OCR SD rats than in OAL animals (Figure 8E), which is in line with better cognitive performances. Similarly, Arc expression was lower in the CA1 region of OAL SD rats compared to other groups (Figure 8F).

### Cognitive performances and anxious behaviors are associated with glutamatergic signaling and stress-related markers

**Figure 9:**
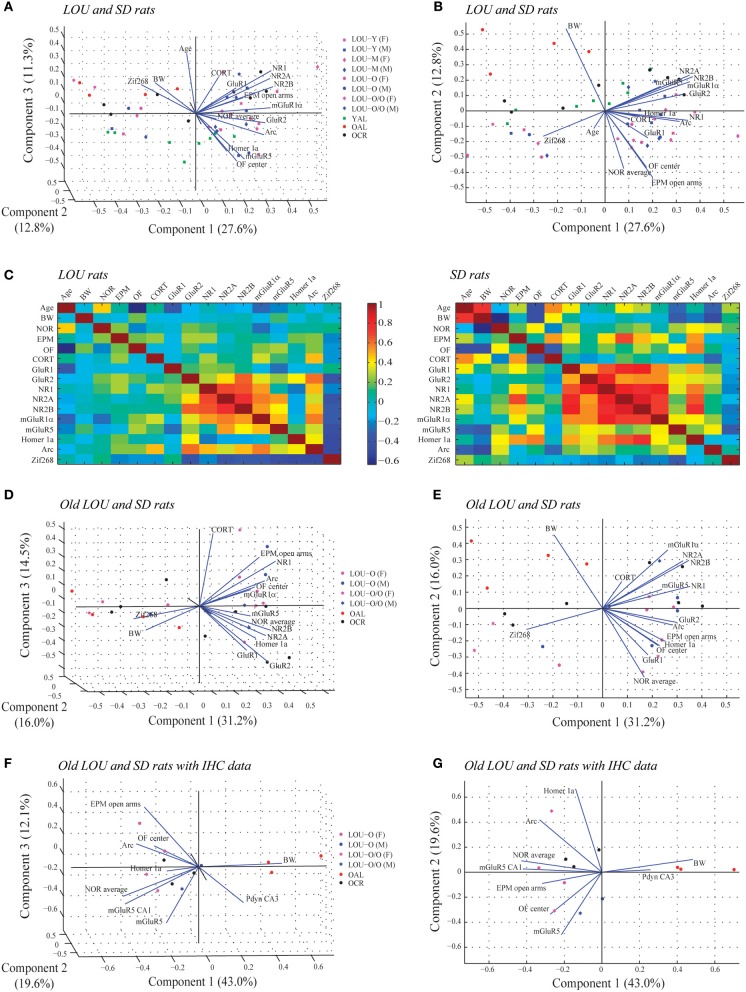
**Associations between physiological parameters, cognition, anxious behaviors, glutamatergic signaling, and stress-related markers in aging LOU and SD rats**. **(A)** Biplot of the principal component coefficients and scores (first three components) of all LOU and SD rats data sets for 16 variables including physiological parameters (age, BW), cognition (NOR average), anxious behaviors (EPM open arms, OF center), stress marker (serum CORT), glutamatergic receptor levels (GluR1, GluR2, NR1, NR2A, NR2B, mGluR1α, mGluR5), and IEG expression (Homer 1a, Arc, Zif268) (*n* = 50, 16 variables). **(B)** Two-dimensional view of the first and second components of **(A)**. **(C)** Correlation matrix for pairs of variables for each rat strain. **(D)** Biplot showing the result of the PCA of old LOU (O, O/O) and SD (OAL, OCR) rats for the 16 variables except age (*n* = 26, 15 variables). **(E)** Two-dimensional view of the first and second component of **(D)**. **(F)** PCA analysis of the 12 rats with IHC data for Pdyn and mGluR5 expression in the hippocampus. Variables include BW as physiological parameter, NOR average for cognition, EPM and OF performances as anxious behaviors, stress-related precursor protein Pdyn and glutamatergic signaling (mGluR5, Homer 1a, Arc) (*n* = 12, 9 variables). **(G)** Two-dimensional view of the first and second component of **(F)**.

We studied the association between physiological parameters (age, BW), cognition (NOR average), anxious behaviors (EPM open arms, OF center), serum CORT, glutamatergic receptors levels (GluR1, GluR2, NR1, NR2A, NR2B, mGluR1α, mGluR5), and IEG expression (Homer 1a, Arc, Zif268) using PCA. When data from all LOU and SD rats were pooled, 51.7% of the overall variance was explained by the first three components (Figure 9A). The main component, corresponding to 27.6% of the variance, was characterized by 13 out of 16 variables on the positive side (Figure 9B), with the exception of age, BW, and Zif268. Next, the impact of rat strain on the correlation matrix was assessed for the 16 variables. As shown on Figure 9C, variation is greater in SD rats then LOU rats. Interestingly, there is a strong association among glutamatergic receptor levels and to some extent between glutamatergic receptor and IEG expression in both rat strains (LOU: positive with Arc, negative with Zif268; SD: positive with Homer 1a, negative with Zif268). In LOU rats, NOR and OF performances were affected by aging while BW had no significant impact. Conversely, BW had a strong negative correlative impact on NOR, OF, mGluR5, and Arc in SD rats. Age also affected serum CORT and ionotropic glutamate receptor levels in this strain. When PCA was repeated using only the aged rodents (O and O/O LOU rats, OAL and OCR SD rats), 61.7% of the overall variance was explained by the first three components (Figure 9D). The main component, corresponding to 31.2% of the variance, was again characterized by all variables being on the positive side, except for BW and Zif268 (Figure 9E). Finally, we tested our conceptual framework using only the 12 rats for which we had IHC data. We studied the association between BW, cognition, anxious behaviors, stress-related precursor protein Pdyn expression in CA3, and glutamatergic signaling (mGluR5, mGluR5 in CA1, Homer 1a and Arc). The first three components of the PCA explained 74.7% of the overall variance with the main component corresponding to 43.0% of the variance (Figure 9F). Increased BW and hippocampal Pdyn expression appears to have a negative effect on behaviors and mGluR5 receptor level and related IEG (Figure 9G). Finally, as shown in Table 1, the main contributors of the first component are BW, cognition, anxious behaviors, Pdyn level, mGluR5 expression in CA1, and IEG Arc, leading us to suggest an association between these variables in healthy aging.

**Table 1:** **Major contributions to the overall variance on the first, second, and third principal components resulting from the principal component analysis (PCA, Figures 9F,G) applied to old and very old male and female LOU rats (*n* = 6) and old SD rats (*n* = 6) with IHC data**.

	**Components**		
	**1st (43.0%)**	**2nd (19.6%)**	**3rd (12.1%)**
**PHYSIOLOGICAL PARAMETER**			
BW	**0.481**	0.101	0.010
**COGNITION**			
NOR average	**−0.425**	0.091	−0.294
**EMOTIONAL BEHAVIORS**			
EPM open arms	**−0.315**	−0.089	**0.592**
OF center	**−0.273**	**−0.334**	**0.262**
**STRESS-RELATED MARKER**			
Pdyn CA3	**0.256**	0.019	**−0.348**
**GLUTAMATERGIC SIGNALING**			
mGluR5	−0.214	**−0.497**	**−0.447**
mGluR5 CA1	**−0.418**	0.026	**−0.357**
Homer 1a	−0.139	**0.663**	−0.155
Arc	**−0.329**	**0.417**	0.150

Higher loading factors (> ±0.25) for each component of the PCA are indicated in bold.

## Discussion

In the present study, obesity-resistant LOU rats were characterized by intact recognition and spatial memory, high levels of hippocampal and EC glutamate receptor and related IEG as well as low anxiety and *Pdyn* expression despite aging. LTCR prevented high BW and favor an increase of glutamate receptor and IEG expression, facilitating memory consolidation. Anxious behaviors were associated with elevated *Pdyn* expression in OAL SD rat hippocampus/EC.

Age-related recognition memory decline is common in animal models and humans (Burke et al., 2012) but somehow remains controversial in rats (Markowska et al., 1998; Shukitt-Hale et al., 2001; Carter et al., 2009). Intact recognition memory was reported in 24-month-old male LOU rats and related to unaltered NMDA-mediated synaptic plasticity in the CA1 hippocampal region (Kollen et al., 2010). Here, the impact of aging on memory was investigated in detail, both in male and female rats from adulthood up to very old age. Since median lifespan of LOU rats is higher than common rat strains (Alliot et al., 2002), memory impairments could be expected to occur at a more advanced age. However, intact recognition memory was observed up to 42 months of age in line with stable expression of glutamate receptor subunits. In addition, we report the performances of aging LOU rats for learning acquisition, reversal learning, and memory retention in the MWM reference task, which is extensively used to discriminate memory-impaired from memory-unimpaired aged rats (Burke et al., 2012; Menard and Quirion, 2012b; Yang et al., 2013). All LOU rats performed well in the MWM task except the 24-month-old male in the learning acquisition task. Nevertheless, improvement was similar to young animals and an extra day of training might be required in future studies to confirm whether or not this function in impaired in old male LOU rats. In contrast, 24-month-old AL male SD rats were shown to develop memory impairments and to exhibit lower NMDA receptor levels and function compared to young rats (Kollen et al., 2010). We observed significant recognition memory impairments in 20-month-old AL SD rats and this deficit was prevented by LTCR. Caloric restriction was shown to facilitate learning consolidation through mechanisms dependent of NMDA receptors in adult mice (Fontan-Lozano et al., 2007). In *F*_344_ × BN rats, LTCR stabilizes age-related decreases of ionotropic glutamate receptors (Adams et al., 2008). Here we report a strong association between AMPA GluR1 and GluR2, NMDA NR1, NR2A, NR2B, and mGluR1α protein levels in SD rats. In contrast, LOU rat correlation matrix revealed an association between NMDA and group 1 mGluR levels and to some extent, GluR2, but not with GluR1. Moreover, GluR1 level was positively associated with cognition and low anxious behaviors in SD but not in LOU rats. NMDA-mediated synaptic plasticity involves efficient AMPA receptor trafficking (Huganir and Nicoll, 2013). However, insertion of GluR1 homomeric receptors in synaptic membranes can lead to AMPA-mediated excitotoxicity (Menard et al., 2007) as GluR2 subunits are recognized to confer calcium impermeability to AMPA receptors (Sommer et al., 1991; Geiger et al., 1995). Strain-related differences in hippocampal learning tasks and regulation of AMPA receptors have been previously reported in mice (Upchurch and Wehner, 1988, 1989; Rossi-Arnaud and Ammassari-Teule, 1998; Menard et al., 2004) but, to our knowledge, not in rats yet. In addition, we found that the memory status of OAL SD rats varies between individuals as well as the impact of LTCR. Variability of age-associated memory decline within the SD rat strain has previously been described and seems to appear only with increasing age (Kollen et al., 2010). Furthermore, individual differences are known to exist in aging rat populations (Zyzak et al., 1995; Menard and Quirion, 2012b). Accordingly, in our study variations are very low for both recognition and spatial memory tasks in young SD but increase with aging. Here, we show that LTCR improved spatial memory performances of old SD rats but did not completely prevent age-related cognitive deficits despite maintenance of youthful NMDA receptor expression. The impact of caloric restriction on cognitive aging seems to be highly dependent of the rat strain and genetic variance (Markowska and Savonenko, 2002).

Group 1 mGluR function and signaling in the hippocampus CA1 and CA3 regions correlates with successful cognitive aging in rats (Lee et al., 2005; Menard and Quirion, 2012b; Yang et al., 2013), and mGluR5^−/−^ mice show impairments in inhibitory learning and spatial memory (Xu et al., 2009). In line with intact memory, mGluR5 expression was maintained throughout life in LOU rats. Interestingly, LTCR prevented the decrease of mGluR5 observed in the hippocampus of old SD rats. A reduction of hypothalamic mGluR5 activation was shown to mediate appetite and BW increase in mGluR5^−/−^ mice (Bradbury et al., 2005). Thus, a lack of mGluR5 might reduce susceptibility to diet-induced insulin resistance, since mGluR5^−/−^ mice show decreased plasma levels of leptin and insulin, despite a 10-week high-fat diet (Bradbury et al., 2005). Improved leptin sensitivity has been reported in the young LOU rat (Veyrat-Durebex et al., 2013) and could be related to unaltered BW despite aging. Our group recently showed that age-related recognition and spatial memory deficits can be reversed following injections of an mGluR5 agonist (Menard et al., 2013). While young adult animals seem to rely on NMDA receptor function to establish and consolidate memories, mGluR function becomes crucial in the old hippocampus (Menard et al., 2013; Yang et al., 2013). Thus, a lower mGluR5 expression in OAL SD rat brain might be a consequence of obesity, affecting memory processes at advanced age.

To our knowledge, this study is also the first one to examine the impact of age-related obesity on the expression of IEG linked to memory formation and consolidation. *Homer 1a* is a dominant negative modulator of group 1 mGluR activity (Kammermeier and Worley, 2007), and its expression is experience-dependent (Nikbakht et al., 2012). Both reduction (Menard and Quirion, 2012b; Kaja et al., 2013) and overexpression (Klugmann et al., 2005) of Homer 1a levels have been associated with memory deficits, highlighting a tightly regulated expression. Here we report a strong association in SD rats between protein levels of Homer 1a, AMPA GluR1, and NMDA receptor subunits. *Homer 1a* modulates the efficacy of synaptic transmission (Inoue et al., 2007) and may facilitate NMDA-mediated synaptic plasticity in aging SD rats. LTCR could help maintaining youthful levels of ionotropic glutamate receptors and Homer 1a, favoring activity-induced synaptic plasticity and then prevent, to some extent, age-related memory-impairments in OCR rats. Interestingly, Homer 1a expression was also positively correlated with exploratory behaviors in the EPM and OF tasks in this strain. Our group recently reported low anxious behaviors and elevated Homer 1a expression in the hippocampus of aged Pdyn KO mice (Menard et al., 2013). The IEG *Arc* is necessary for memory consolidation and successful cognitive aging in rodents (Penner et al., 2011; Menard and Quirion, 2012b; Menard et al., 2013), and post-training disruption of Arc expression impairs long-term memory (Holloway and McIntyre, 2011). LTCR prevented age-related reduction of Homer 1a and Arc expression following behavioral training in SD rats, in line with better cognitive performances. LOU rats were characterized by a strong association between NMDA receptor subunits, mGluR5 and Arc levels which were all negatively correlated to Zif268 expression. Intact NMDA-mediated synaptic plasticity was reported in the CA1 hippocampal region of 24-month-old LOU rats (Kollen et al., 2010). On the other hand, group 1 mGluR-mediated synaptic plasticity has been associated with successful cognitive aging (Menard et al., 2013; Yang et al., 2013), formation of spatial (Lu et al., 1997; Balschun et al., 1999; Menard and Quirion, 2012b; Menard et al., 2013) and recognition memory (Barker et al., 2006; Christoffersen et al., 2008; Menard et al., 2013). Maintenance of both forms of synaptic plasticity in aging LOU rats could protect memory function up to a very advanced age. Following neuronal activity, Arc encoding mRNA is quickly transported to the dendrites and then translated (Bramham et al., 2008; Shepherd and Bear, 2011). This IEG has been associated to synapse-specific homeostatic plasticity (Beique et al., 2011) which is crucial for learning and memory processes and seems to be intact in the aging LOU rats. Strong association of Arc with glutamatergic receptor levels may explain better performances in the MWM reversal learning task for the LOU rat groups. Indeed, expression of this IEG is positively correlated with hippocampus-dependent spatial reversal learning (Guzowski et al., 2001). While the role of Arc in synaptic plasticity may cover several phases of memory formation, Zif268 is more important in the early steps of memory consolidation (Alberini, 2009). Prolonged training might decrease its expression in memory-unimpaired animals, such as the LOU rats. Interestingly, Zif268 expression is negatively correlated with mGluR5 in both rat strains, suggesting a strong interaction between those in successful cognitive processes in the aged brain.

Obesity-resistant LOU rats were also characterized by low hippocampal and EC Pdyn expression and stable circulating CORT. Low CORT has been reported in 20-week-old LOU rats (Veyrat-Durebex et al., 2009). We found that serum CORT levels were higher in 12-month-old and older male rats than young ones but remained stable in females. This sexual dimorphism may explain, at least in part, the differences observed in spatial learning acquisition and reverse probe test. Older LOU rats may also be less sensitive to CORT than other rat strains (Marissal-Arvy et al., 2007) which would dampen stress-induced learning and memory deficits. Caloric restriction increases CORT release in adult (Levay et al., 2010) and old animals (Bedard et al., 2010). Beneficial effects provided by dietary restriction can be enhanced by reducing GC production (Qiu et al., 2012), suggesting that lowering circulating CORT in old SD rats may help preventing spatial memory impairments. Anxiety-related behaviors in the EPM task can be reduced after 8 days of caloric restriction in adult mice (Yamamoto et al., 2009). Here, we provide the first evidence that age-related exacerbated anxiety can be prevented by LTCR. Short-term food deprivation reduces Pdyn level in the arcuate nucleus (Kim et al., 1996), and we found that LTCR significantly decreases this stress-related peptide expression in the aged hippocampus. Therefore, LOU rats might be more resilient to stressful events (Russo et al., 2012). Resilience has been associated with increased lifespan (Vahia et al., 2011), which is a characteristic of this rat strain, as shown by Alliot et al. (2002) and extended here.

Stress and elevated circulating CORT can induce cognitive impairments (Landfield et al., 1981; Bodnoff et al., 1995; Lupien et al., 2005; Sandi, 2011). Enhanced serum CORT were found in naïve 20-month-old CR SD rats but not in age-matched AL rats (Bedard et al., 2010), raising the hypothesis that behavioral training might promote sustained threat perception in OAL SD rats, exacerbating basal levels of stress response effectors (O'Donovan et al., 2013). Accordingly, *Pdyn* expression was significantly increased in OAL hippocampus and EC. Activation of κ-opioid receptors (KOR), the main target of dynorphins, suppresses glutamate release (Simmons et al., 1994), and KOR activation mediates stress-induced memory deficits (Carey et al., 2009). Reduction of *Pdyn* gene expression protects old mice from cognitive impairments and anxious behaviors through upregulation of group 1 mGluR expression and function (Menard et al., 2013) and may favor AMPA GluR1 and NMDA NR2B expression in the aged brain (Menard et al., 2014). Both group 1 mGluR function and *Homer 1* expression have been associated with stress-induced cognitive deficits (Tronson et al., 2010; Menard et al., 2013; Wagner et al., 2013), and anxious behaviors are exacerbated by aging in the mGluR5^−/−^ mice (Inta et al., 2013). Age-related obesity, as observed in OAL SD rats, could affect multiple neurotransmitters and modulators leading to exacerbated anxiety and memory deficits. In contrast, LOU rats may be protected from these alterations through a better regulation of brain plasticity-related genes during aging (Paban et al., 2012).

In summary, intact recognition and spatial memory in the obese-resistant LOU rat strain is associated with low anxiety responses, low levels of the stress-related *Pdyn* in the hippocampus and EC and enhanced expression of synaptic plasticity effectors at the cellular and molecular level. On the other hand, age-related weight gain affects lifespan, glutamate receptors and IEG expression, exacerbates anxiety and induced memory deficits. Nutritional intervention such as adult-onset LTCR can prevent age-induced reduction of glutamate receptor expression and signaling in SD rats, in line with the results obtained for the LOU rats but does not allow to completely preserve youthful spatial memory. Future studies will therefore be necessary to confirm if LTCR has the same effect on other rat strains including Wistar rats since LOU rats are thought to be of Wistar origin (Alliot et al., 2002). Whole genome or exome sequencing of the LOU rat will also have to be performed to allow determining if this strain is indeed originating from the Wistar stain. Another set of interesting experiments would be to evaluate if high-fat diet can induce obesity in aging LOU rats and affect cognition and/or lifespan. Finally, whether or not preservation of healthy memory functions in the aging LOU rat is related to a general or tissue-specific mechanism will deserve further attention. However, our results support a novel conceptual framework and highlight potential pharmacological targets to treat cognitive decline and anxious behaviors associated with aging-related obesity.

## Author contributions

Caroline Ménard, Rémi Quirion, Guylaine Ferland, and Pierrette Gaudreau designed research; Caroline Ménard performed research; Caroline Ménard and Sylvain Bouchard analyzed data; Caroline Ménard, Sylvain Bouchard, and Pierrette Gaudreau interpreted the data; Caroline Ménard, Rémi Quirion, Guylaine Ferland, and Pierrette Gaudreau wrote the paper.

### Conflict of interest statement

Animal protocols were approved by the CHUM Research Center and the University of Montreal Animal Care Committees in compliance with Canadian Council for Animal Care guidelines. The authors declare that the research was conducted in the absence of any commercial or financial relationships that could be construed as a potential conflict of interest.

## References

[B1] Adams M. M. Shi L. Linville M. C. Forbes M. E. Long A. B. Bennett C. (2008). Caloric restriction and age affect synaptic proteins in hippocampal CA3 and spatial learning ability. Exp. Neurol. 211, 141–149 10.1016/j.expneurol.2008.01.016 18342310 PMC2805131

[B2] Alberini C. M. (2009). Transcription factors in long-term memory and synaptic plasticity. Physiol. Rev. 89, 121–145 10.1152/physrev.00017.2008 19126756 PMC3883056

[B3] Alliot J. Boghossian S. Jourdan D. Veyrat-Durebex C. Pickering G. Meynial-Denis D. (2002). The LOU/c/jall rat as an animal model of healthy aging? J. Gerontol. A Biol. Sci. Med. Sci. 57, B312–B320 10.1093/gerona/57.8.B312 12145357

[B4] Balschun D. Manahan-Vaughan D. Wagner T. Behnisch T. Reymann K. G. Wetzel W. (1999). A specific role for group I mGluRs in hippocampal LTP and hippocampus-dependent spatial learning. Learn. Mem. 6, 138–152 10.1101/lm.6.2.138 10327239 PMC311286

[B5] Barker G. R. Bashir Z. I. Brown M. W. Warburton E. C. (2006). A temporally distinct role for group I and group II metabotropic glutamate receptors in object recognition memory. Learn. Mem. 13, 178–186 10.1101/lm.77806 16585793 PMC1409835

[B6] Baudry M. Bi X. Gall C. Lynch G. (2011). The biochemistry of memory: the 26year journey of a ‘new and specific hypothesis.’ Neurobiol. Learn. Mem. 95, 125–133 10.1016/j.nlm.2010.11.015 21134478 PMC3042723

[B7] Bedard K. Bedard J. Rocheleau G. Ferland G. Gaudreau P. (2013). Aging and diets regulate the rat anterior pituitary and hypothalamic transcriptome. Neuroendocrinology 97, 146–159 10.1159/000338411 22538389

[B8] Bedard K. Robinette K. Ferland G. Gaudreau P. (2010). Effects of long-term dietary interventions on pituitary growth hormone-releasing hormone receptor in aging rats and potential mechanisms of action. Mech. Ageing Dev. 131, 169–178 10.1016/j.mad.2010.01.003 20122951

[B9] Beique J. C. Na Y. Kuhl D. Worley P. F. Huganir R. L. (2011). Arc-dependent synapse-specific homeostatic plasticity. Proc. Natl. Acad. Sci. U.S.A. 108, 816–821 10.1073/pnas.1017914108 21187403 PMC3021034

[B10] Benoit C. E. Rowe W. B. Menard C. Sarret P. Quirion R. (2011). Genomic and proteomic strategies to identify novel targets potentially involved in learning and memory. Trends Pharmacol. Sci. 32, 43–52 10.1016/j.tips.2010.10.002 21129790

[B11] Bilkei-Gorzo A. Erk S. Schurmann B. Mauer D. Michel K. Boecker H. (2012). Dynorphins regulate fear memory: from mice to men. J. Neurosci. 32, 9335–9343 10.1523/JNEUROSCI.1034-12.2012 22764240 PMC6622224

[B12] Bodnoff S. R. Humphreys A. G. Lehman J. C. Diamond D. M. Rose G. M. Meaney M. J. (1995). Enduring effects of chronic corticosterone treatment on spatial learning, synaptic plasticity, and hippocampal neuropathology in young and mid-aged rats. J. Neurosci. 15, 61–69 7823152 10.1523/JNEUROSCI.15-01-00061.1995 PMC6578287

[B13] Bradbury M. J. Campbell U. Giracello D. Chapman D. King C. Tehrani L. (2005). Metabotropic glutamate receptor mGlu5 is a mediator of appetite and energy balance in rats and mice. J. Pharmacol. Exp. Ther. 313, 395–402 10.1124/jpet.104.076406 15590770

[B14] Bramham C. R. Worley P. F. Moore M. J. Guzowski J. F. (2008). The immediate early gene arc/arg3.1: regulation, mechanisms, and function. J. Neurosci. 28, 11760–11767 10.1523/JNEUROSCI.3864-08.2008 19005037 PMC2615463

[B15] Brim B. L. Haskell R. Awedikian R. Ellinwood N. M. Jin L. Kumar A. (2012). Memory in aged mice is rescued by enhanced expression of the GluN2B subunit of the NMDA receptor. Behav. Brain Res. 238C, 211–226 10.1016/j.bbr.2012.10.026 23103326 PMC3540206

[B16] Bunce D. Batterham P. J. Mackinnon A. J. Christensen H. (2012). Depression, anxiety and cognition in community-dwelling adults aged 70 years and over. J. Psychiatr. Res. 46, 1662–1666 10.1016/j.jpsychires.2012.08.023 23017811

[B17] Burke S. N. Ryan L. Barnes C. A. (2012). Characterizing cognitive aging of recognition memory and related processes in animal models and in humans. Front. Aging Neurosci. 4:15 10.3389/fnagi.2012.00015 22988437 PMC3439640

[B18] Burke S. N. Wallace J. L. Hartzell A. L. Nematollahi S. Plange K. Barnes C. A. (2011). Age-associated deficits in pattern separation functions of the perirhinal cortex: a cross-species consensus. Behav. Neurosci. 125, 836–847 10.1037/a0026238 22122147 PMC3255096

[B19] Carey A. N. Lyons A. M. Shay C. F. Dunton O. McLaughlin J. P. (2009). Endogenous kappa opioid activation mediates stress-induced deficits in learning and memory. J. Neurosci. 29, 4293–4300 10.1523/JNEUROSCI.6146-08.2009 19339623 PMC6665365

[B20] Carter C. S. Leeuwenburgh C. Daniels M. Foster T. C. (2009). Influence of calorie restriction on measures of age-related cognitive decline: role of increased physical activity. J. Gerontol. A Biol. Sci. Med. Sci. 64, 850–859 10.1093/gerona/glp060 19420296 PMC2709546

[B21] Christoffersen G. R. Simonyi A. Schachtman T. R. Clausen B. Clement D. Bjerre V. K. (2008). MGlu5 antagonism impairs exploration and memory of spatial and non-spatial stimuli in rats. Behav. Brain Res. 191, 235–245 10.1016/j.bbr.2008.03.032 18471908

[B22] Czerniawski J. Ree F. Chia C. Ramamoorthi K. Kumata Y. Otto T. A. (2011). The importance of having Arc: expression of the immediate-early gene Arc is required for hippocampus-dependent fear conditioning and blocked by NMDA receptor antagonism. J. Neurosci. 31, 11200–11207 10.1523/JNEUROSCI.2211-11.2011 21813681 PMC6623359

[B23] Fadel J. R. Jolivalt C. G. Reagan L. P. (2013). Food for thought: the role of appetitive peptides in age-related cognitive decline. Ageing Res. Rev. 12, 764–776 10.1016/j.arr.2013.01.009 23416469 PMC3774057

[B24] Fontan-Lozano A. Saez-Cassanelli J. L. Inda M. C. De Los Santos-Arteaga M. Sierra-Dominguez S. A. Lopez-Lluch G. (2007). Caloric restriction increases learning consolidation and facilitates synaptic plasticity through mechanisms dependent on NR2B subunits of the NMDA receptor. J. Neurosci. 27, 10185–10195 10.1523/JNEUROSCI.2757-07.2007 17881524 PMC6672666

[B25] Frankland P. W. Bontempi B. (2005). The organization of recent and remote memories. Nat. Rev. Neurosci. 6, 119–130 10.1038/nrn1607 15685217

[B26] Geiger J. R. Melcher T. Koh D. S. Sakmann B. Seeburg P. H. Jonas P. (1995). Relative abundance of subunit mRNAs determines gating and Ca2+ permeability of AMPA receptors in principal neurons and interneurons in rat CNS. Neuron 15, 193–204 10.1016/0896-6273(95)90076-4 7619522

[B27] Guzowski J. F. Setlow B. Wagner E. K. McGaugh J. L. (2001). Experience-dependent gene expression in the rat hippocampus after spatial learning: a comparison of the immediate-early genes Arc, c-fos, and zif268. J. Neurosci. 21, 5089–5098 11438584 10.1523/JNEUROSCI.21-14-05089.2001 PMC6762831

[B28] Harrison F. E. Hosseini A. H. McDonald M. P. (2009). Endogenous anxiety and stress responses in water maze and Barnes maze spatial memory tasks. Behav. Brain Res. 198, 247–251 10.1016/j.bbr.2008.10.015 18996418 PMC2663577

[B29] Holloway C. M. McIntyre C. K. (2011). Post-training disruption of Arc protein expression in the anterior cingulate cortex impairs long-term memory for inhibitory avoidance training. Neurobiol. Learn. Mem. 95, 425–432 10.1016/j.nlm.2011.02.002 21315825

[B30] Huganir R. L. Nicoll R. A. (2013). AMPARs and synaptic plasticity: the last 25 years. Neuron 80, 704–717 10.1016/j.neuron.2013.10.025 24183021 PMC4195488

[B31] Inoue N. Nakao H. Migishima R. Hino T. Matsui M. Hayashi F. (2009). Requirement of the immediate early gene vesl-1S/homer-1a for fear memory formation. Mol. Brain 2, 7 10.1186/1756-6606-2-7 19265511 PMC2663561

[B32] Inoue Y. Udo H. Inokuchi K. Sugiyama H. (2007). Homer1a regulates the activity-induced remodeling of synaptic structures in cultured hippocampal neurons. Neuroscience 150, 841–852 10.1016/j.neuroscience.2007.09.081 18006237

[B33] Inta D. Vogt M. A. Luoni A. Filipovic D. Lima-Ojeda J. M. Pfeiffer N. (2013). Significant increase in anxiety during aging in mGlu5 receptor knockout mice. Behav. Brain Res. 241, 27–31 10.1016/j.bbr.2012.11.042 23228523

[B34] Kaja S. Sumien N. Borden P. K. Khullar N. Iqbal M. Collins J. L. (2013). Homer-1a immediate early gene expression correlates with better cognitive performance in aging. Age 35, 1799–1808 10.1007/s11357-012-9479-6 23054826 PMC3776093

[B35] Kammermeier P. J. Worley P. F. (2007). Homer 1a uncouples metabotropic glutamate receptor 5 from postsynaptic effectors. Proc. Natl. Acad. Sci. U.S.A. 104, 6055–6060 10.1073/pnas.0608991104 17389377 PMC1851615

[B36] Kesner R. P. (2007). Behavioral functions of the CA3 subregion of the hippocampus. Learn. Mem. 14, 771–781 10.1101/lm.688207 18007020

[B37] Kim E. M. Welch C. C. Grace M. K. Billington C. J. Levine A. S. (1996). Chronic food restriction and acute food deprivation decrease mRNA levels of opioid peptides in arcuate nucleus. Am. J. Physiol. 270, R1019–R1024 8928900 10.1152/ajpregu.1996.270.5.R1019

[B38] Klugmann M. Symes C. W. Leichtlein C. B. Klaussner B. K. Dunning J. Fong D. (2005). AAV-mediated hippocampal expression of short and long Homer 1 proteins differentially affect cognition and seizure activity in adult rats. Mol. Cell. Neurosci. 28, 347–360 10.1016/j.mcn.2004.10.002 15691715

[B39] Kollen M. Stephan A. Faivre-Bauman A. Loudes C. Sinet P. M. Alliot J. (2010). Preserved memory capacities in aged Lou/C/Jall rats. Neurobiol. Aging 31, 129–142 10.1016/j.neurobiolaging.2008.03.010 18462838

[B40] Kotz C. M. Weldon D. Billington C. J. Levine A. S. (2004). Age-related changes in brain proDynorphin gene expression in the rat. Neurobiol. Aging 25, 1343–1347 10.1016/j.neurobiolaging.2004.02.025 15465632

[B41] Landfield P. W. Baskin R. K. Pitler T. A. (1981). Brain aging correlates: retardation by hormonal-pharmacological treatments. Science 214, 581–584 10.1126/science.6270791 6270791

[B42] Lee H. K. Min S. S. Gallagher M. Kirkwood A. (2005). NMDA receptor-independent long-term depression correlates with successful aging in rats. Nat. Neurosci. 8, 1657–1659 10.1038/nn1586 16286930

[B43] Levay E. A. Tammer A. H. Penman J. Kent S. Paolini A. G. (2010). Calorie restriction at increasing levels leads to augmented concentrations of corticosterone and decreasing concentrations of testosterone in rats. Nutr. Res. 30, 366–373 10.1016/j.nutres.2010.05.001 20579529

[B44] Lu Y. M. Jia Z. Janus C. Henderson J. T. Gerlai R. Wojtowicz J. M. (1997). Mice lacking metabotropic glutamate receptor 5 show impaired learning and reduced CA1 long-term potentiation (LTP) but normal CA3 LTP. J. Neurosci. 17, 5196–5205 9185557 10.1523/JNEUROSCI.17-13-05196.1997 PMC6573299

[B45] Lupien S. J. Fiocco A. Wan N. Maheu F. Lord C. Schramek T. (2005). Stress hormones and human memory function across the lifespan. Psychoneuroendocrinology 30, 225–242 10.1016/j.psyneuen.2004.08.003 15511597

[B46] Lupien S. J. McEwen B. S. Gunnar M. R. Heim C. (2009). Effects of stress throughout the lifespan on the brain, behaviour and cognition. Nat. Rev. Neurosci. 10, 434–445 10.1038/nrn2639 19401723

[B47] Marissal-Arvy N. Gaumont A. Langlois A. Dabertrand F. Bouchecareilh M. Tridon C. (2007). Strain differences in hypothalamic pituitary adrenocortical axis function and adipogenic effects of corticosterone in rats. J. Endocrinol. 195, 473–484 10.1677/JOE-07-0077 18000309

[B48] Markowska A. L. Mooney M. Sonntag W. E. (1998). Insulin-like growth factor-1 ameliorates age-related behavioral deficits. Neuroscience 87, 559–569 10.1016/S0306-4522(98)00143-2 9758223

[B49] Markowska A. L. Savonenko A. (2002). Retardation of cognitive aging by life-long diet restriction: implications for genetic variance. Neurobiol. Aging 23, 75–86 10.1016/S0197-4580(01)00249-4 11755022

[B50] Mathus-Vliegen E. M. (2012). Obesity and the elderly. J. Clin. Gastroenterol. 46, 533–544 10.1097/MCG.0b013e31825692ce 22772735

[B51] Menard C. Chartier E. Patenaude C. Robinson P. Cyr M. Baudry M. (2007). Calcium-independent phospholipase A(2) influences AMPA-mediated toxicity of hippocampal slices by regulating the GluR1 subunit in synaptic membranes. Hippocampus 17, 1109–1120 10.1002/hipo.20343 17696174

[B52] Menard C. Herzog H. Schwarzer C. Quirion R. (2014). Possible role of dynorphins in Alzheimer's disease and age-related cognitive deficits. Neurodegener. Dis. 13, 82–85 10.1159/000353848 23970097

[B53] Menard C. Quirion R. (2012a). Group 1 metabotropic glutamate receptor function and its regulation of learning and memory in the aging brain. Front. Pharmacol. 3:182 10.3389/fphar.2012.00182 23091460 PMC3469824

[B54] Menard C. Quirion R. (2012b). Successful cognitive aging in rats: a role for mGluR5 glutamate receptors, homer 1 proteins and downstream signaling pathways. PLoS ONE 7:e28666 10.1371/journal.pone.0028666 22238580 PMC3253083

[B55] Menard C. Tse Y. C. Cavanagh C. Chabot J. G. Herzog H. Schwarzer C. (2013). Knockdown of prodynorphin gene prevents cognitive decline, reduces anxiety, and rescues loss of group 1 metabotropic glutamate receptor function in aging. J. Neurosci. 33, 12792–12804 10.1523/JNEUROSCI.0290-13.2013 23904614 PMC6618545

[B56] Menard C. Valastro B. Martel M. A. Martinoli M. G. Massicotte G. (2004). Strain-related variations of AMPA receptor modulation by calcium-dependent mechanisms in the hippocampus: contribution of lipoxygenase metabolites of arachidonic acid. Brain Res. 1010, 134–143 10.1016/j.brainres.2004.03.012 15126126

[B57] Moura P. J. Meirelles S. T. Xavier G. F. (2010). Long-term social recognition memory in adult male rats: factor analysis of the social and non-social behaviors. Braz. J. Med. Biol. Res. 43, 663–676 10.1590/S0100-879X2010007500047 20512300

[B58] Moyse E. Bedard K. Segura S. Mahaut S. Tardivel C. Ferland G. (2012). Effects of aging and caloric restriction on brainstem satiety center signals in rats. Mech. Ageing Dev. 133, 83–91 10.1016/j.mad.2012.01.004 22285292

[B59] Newmark R. E. Schon K. Ross R. S. Stern C. E. (2013). Contributions of the hippocampal subfields and entorhinal cortex to disambiguation during working memory. Hippocampus 23, 467–475 10.1002/hipo.22106 23504938 PMC4419744

[B60] Nguyen X. V. Masse J. Kumar A. Vijitruth R. Kulik C. Liu M. (2005). Prodynorphin knockout mice demonstrate diminished age-associated impairment in spatial water maze performance. Behav. Brain Res. 161, 254–262 10.1016/j.bbr.2005.02.010 15922052

[B61] Nikbakht N. Zarei B. Shirani E. Moshtaghian J. Esmaeili A. Habibian S. (2012). Experience-dependent expression of rat hippocampal Arc and Homer 1a after spatial learning on 8-arm and 12-arm radial mazes. Neuroscience 218, 49–55 10.1016/j.neuroscience.2012.05.025 22617701

[B62] O'Donovan A. Slavich G. M. Epel E. S. Neylan T. C. (2013). Exaggerated neurobiological sensitivity to threat as a mechanism linking anxiety with increased risk for diseases of aging. Neurosci. Biobehav. Rev. 37, 96–108 10.1016/j.neubiorev.2012.10.013 23127296 PMC4361087

[B63] Paban V. Billard J. M. Bouet V. Freret T. Boulouard M. Chambon C. (2012). Genomic transcriptional profiling in LOU/C/Jall rats identifies genes for successful aging. Brain Struct. Funct. 218, 1501–1512 10.1007/s00429-012-0472-8 23143343

[B64] Penner M. R. Roth T. L. Chawla M. K. Hoang L. T. Roth E. D. Lubin F. D. (2011). Age-related changes in Arc transcription and DNA methylation within the hippocampus. Neurobiol. Aging 32, 2198–2210 10.1016/j.neurobiolaging.2010.01.009 20189687 PMC2888808

[B65] Qiu G. Spangler E. L. Wan R. Miller M. Mattson M. P. So K. F. (2012). Neuroprotection provided by dietary restriction in rats is further enhanced by reducing glucocortocoids. Neurobiol. Aging 33, 2398–2410 10.1016/j.neurobiolaging.2011.11.025 22226488 PMC3374050

[B66] Rossi-Arnaud C. Ammassari-Teule M. (1998). What do comparative studies of inbred mice add to current investigations on the neural basis of spatial behaviors? Exp. Brain Res. 123, 36–44 10.1007/s002210050542 9835390

[B67] Rowe W. B. Blalock E. M. Chen K. C. Kadish I. Wang D. Barrett J. E. (2007). Hippocampal expression analyses reveal selective association of immediate-early, neuroenergetic, and myelinogenic pathways with cognitive impairment in aged rats. J. Neurosci. 27, 3098–3110 10.1523/JNEUROSCI.4163-06.2007 17376971 PMC6672456

[B68] Russo S. J. Murrough J. W. Han M. H. Charney D. S. Nestler E. J. (2012). Neurobiology of resilience. Nat. Neurosci. 15, 1475–1484 10.1038/nn.3234 23064380 PMC3580862

[B69] Sandi C. (2011). Glucocorticoids act on glutamatergic pathways to affect memory processes. Trends Neurosci. 34, 165–176 10.1016/j.tins.2011.01.006 21377221

[B70] Schwarzer C. (2009). 30 years of dynorphins–new insights on their functions in neuropsychiatric diseases. Pharmacol. Ther. 123, 353–370 10.1016/j.pharmthera.2009.05.006 19481570 PMC2872771

[B71] Shepherd J. D. Bear M. F. (2011). New views of Arc, a master regulator of synaptic plasticity. Nat. Neurosci. 14, 279–284 10.1038/nn.2708 21278731 PMC8040377

[B72] Shukitt-Hale B. Casadesus G. Cantuti-Castelvetri I. Joseph J. A. (2001). Effect of age on object exploration, habituation, and response to spatial and nonspatial change. Behav. Neurosci. 115, 1059–1064 10.1037//0735-7044.115.5.1059 11584918

[B73] Simmons M. L. Terman G. W. Drake C. T. Chavkin C. (1994). Inhibition of glutamate release by presynaptic kappa 1-opioid receptors in the guinea pig dentate gyrus. J. Neurophysiol. 72, 1697–1705 7823095 10.1152/jn.1994.72.4.1697

[B74] Sommer B. Kohler M. Sprengel R. Seeburg P. H. (1991). RNA editing in brain controls a determinant of ion flow in glutamate-gated channels. Cell 67, 11–19 10.1016/0092-8674(91)90568-J 1717158

[B75] Stranahan A. M. Mattson M. P. (2011). Bidirectional metabolic regulation of neurocognitive function. Neurobiol. Learn. Mem. 96, 507–516 10.1016/j.nlm.2011.01.004 21236352 PMC3084367

[B76] Traissard N. Herbeaux K. Cosquer B. Jeltsch H. Ferry B. Galani R. (2007). Combined damage to entorhinal cortex and cholinergic basal forebrain neurons, two early neurodegenerative features accompanying Alzheimer's disease: effects on locomotor activity and memory functions in rats. Neuropsychopharmacology 32, 851–871 10.1038/sj.npp.1301116 16760925

[B77] Tronson N. C. Guzman Y. F. Guedea A. L. Huh K. H. Gao C. Schwarz M. K. (2010). Metabotropic glutamate receptor 5/Homer interactions underlie stress effects on fear. Biol. Psychiatry 68, 1007–1015 10.1016/j.biopsych.2010.09.004 21075228 PMC2987592

[B78] Umegaki H. Hayashi T. Nomura H. Yanagawa M. Nonogaki Z. Nakshima H. (2013). Cognitive dysfunction: an emerging concept of a new diabetic complication in the elderly. Geriatr. Gerontol. Int. 13, 28–34 10.1111/j.1447-0594.2012.00922.x 22882533

[B79] Upchurch M. Wehner J. M. (1988). DBA/2Ibg mice are incapable of cholinergically-based learning in the Morris water task. Pharmacol. Biochem. Behav. 29, 325–329 10.1016/0091-3057(88)90164-5 3362927

[B80] Upchurch M. Wehner J. M. (1989). Inheritance of spatial learning ability in inbred mice: a classical genetic analysis. Behav. Neurosci. 103, 1251–1258 10.1037/0735-7044.103.6.1251 2610918

[B81] Vahia I. V. Chattillion E. Kavirajan H. Depp C. A. (2011). Psychological protective factors across the lifespan: implications for psychiatry. Psychiatr. Clin. North Am. 34, 231–248 10.1016/j.psc.2010.11.011 21333850

[B82] Veyrat-Durebex C. Alliot J. Gaudreau P. (2005). Regulation of the pituitary growth hormone-releasing hormone receptor in ageing male and female LOU rats: new insights into healthy ageing. J. Neuroendocrinol. 17, 691–700 10.1111/j.1365-2826.2005.01343.x 16218997

[B83] Veyrat-Durebex C. Montet X. Vinciguerra M. Gjinovci A. Meda P. Foti M. (2009). The Lou/C rat: a model of spontaneous food restriction associated with improved insulin sensitivity and decreased lipid storage in adipose tissue. Am. J. Physiol. Endocrinol. Metab. 296, E1120–E1132 10.1152/ajpendo.90592.2008 19208855

[B84] Veyrat-Durebex C. Poher A. L. Caillon A. Somm E. Vallet P. Charnay Y. (2013). Improved leptin sensitivity as a potential candidate responsible for the spontaneous food restriction of the lou/c rat. PLoS ONE 8:e73452 10.1371/journal.pone.0073452 24039946 PMC3765307

[B85] Wagner J. J. Terman G. W. Chavkin C. (1993). Endogenous dynorphins inhibit excitatory neurotransmission and block LTP induction in the hippocampus. Nature 363, 451–454 10.1038/363451a0 8099201 PMC2096733

[B86] Wagner K. V. Hartmann J. Mangold K. Wang X. D. Labermaier C. Liebl C. (2013). Homer1 mediates acute stress-induced cognitive deficits in the dorsal hippocampus. J. Neurosci. 33, 3857–3864 10.1523/JNEUROSCI.4333-12.2013 23447597 PMC6619309

[B87] Whittington R. A. Bretteville A. Virag L. Emala C. W. Maurin T. O. Marcouiller F. (2013). Anesthesia-induced hypothermia mediates decreased ARC gene and protein expression through ERK/MAPK inactivation. Sci. Rep. 3, 1388 10.1038/srep01388 24045785 PMC3965357

[B88] Williamson R. McNeilly A. Sutherland C. (2012). Insulin resistance in the brain: an old-age or new-age problem? Biochem. Pharmacol. 84, 737–745 10.1016/j.bcp.2012.05.007 22634336

[B89] Wittmann W. Schunk E. Rosskothen I. Gaburro S. Singewald N. Herzog H. (2009). Prodynorphin-derived peptides are critical modulators of anxiety and regulate neurochemistry and corticosterone. Neuropsychopharmacology 34, 775–785 10.1038/npp.2008.142 18800067 PMC2873573

[B90] Xu J. Zhu Y. Contractor A. Heinemann S. F. (2009). mGluR5 has a critical role in inhibitory learning. J. Neurosci. 29, 3676–3684 10.1523/JNEUROSCI.5716-08.2009 19321764 PMC2746052

[B91] Yamamoto Y. Tanahashi T. Kawai T. Chikahisa S. Katsuura S. Nishida K. (2009). Changes in behavior and gene expression induced by caloric restriction in C57BL/6 mice. Physiol. Genomics 39, 227–235 10.1152/physiolgenomics.00082.2009 19737990

[B92] Yang S. Megill A. Ardiles A. O. Ransom S. Tran T. Koh M. T. (2013). Integrity of mGluR-LTD in the Associative/Commissural Inputs to CA3 Correlates with Successful Aging in Rats. J. Neurosci. 33, 12670–12678 10.1523/JNEUROSCI.1086-13.2013 23904603 PMC3728684

[B93] Zhao X. Rosenke R. Kronemann D. Brim B. Das S. R. Dunah A. W. (2009). The effects of aging on N-methyl-D-aspartate receptor subunits in the synaptic membrane and relationships to long-term spatial memory. Neuroscience 162, 933–945 10.1016/j.neuroscience.2009.05.018 19446010 PMC2769499

[B94] Zyzak D. R. Otto T. Eichenbaum H. Gallagher M. (1995). Cognitive decline associated with normal aging in rats: a neuropsychological approach. Learn. Mem. 2, 1–16 10.1101/lm.2.1.1 10467562

