# Supplementary material for: Glutamatergic signaling and low prodynorphin expression are associated with intact memory and reduced anxiety in rat models of healthy aging
Source: Front Aging Neurosci. 2014 May 7;6:81. doi: 10.3389/fnagi.2014.00081 (PMC4019859; doi:10.3389/fnagi.2014.00081)
Supplement: Supplementary file 1 [file DataSheet1.DOCX]

**Table 1.** Physiological parameters of LOU and SD rats

Group Age (months) Sex Diet Body weight (g) CORT (ng/mL)

LOU 6 M AL 272.8 ± 3.9 60.7 ± 33.1

LOU 6 F AL 169.0 ± 1.4 79.1 ± 19.4

LOU 12 M AL 326.0 ± 8.4^a^ 73.7 ± 23.3

LOU 12 F AL 191.4 ± 4.0^b^ 315.0 ± 83.2

LOU 24 M AL 338.6 ± 12.8^a^ 251.2 ± 53.5^g^

LOU 24 F AL 181.0 ± 4.1^c^ 134.6 ± 13.8

LOU 38 M AL 319.0 ± 31.2 170.0 ± 19.4

LOU 38-42 F AL 186.4 ± 4.5 154.8 ± 49.8

SD 3 M AL 474.4 ± 4.9 37.8 ± 9.5

SD 20 M AL 1025.8 ± 61.1^d,f^ 132.1 ± 63.3

SD 20 M CR 612.3 ± 23.8^e^ 155.1 ± 38.8

Values represent the mean ± SEM of 4-11 rats.

^a^ *p* < 0.01 when compared to 6-month-old male LOU rats.

^b^ *p* < 0.01 when compared to 6-month-old female LOU rats.

^c^ *p* < 0.05 when compared to 6-month-old female LOU rats.

^d^ *p* < 0.001 when compared to 3-month-old male SD rats.

^e^ *p* < 0.01 when compared to 3-month-old male SD rats.

^f^ *p* < 0.001 when compared to 20-month-old calorie-restricted male SD rats.

^g^ *p* < 0.05 when compared to 6-month-old male LOU rats.
